# *N*^4^-acetyldeoxycytosine DNA modification marks euchromatin regions in *Arabidopsis thaliana*

**DOI:** 10.1186/s13059-021-02578-7

**Published:** 2022-01-03

**Authors:** Shuai Wang, Hairong Xie, Fei Mao, Haiyan Wang, Shu Wang, Zhenglin Chen, Yuxia Zhang, Zhihui Xu, Jinming Xing, Zhaokang Cui, Xiquan Gao, Hongmei Jin, Jian Hua, Bo Xiong, Yufeng Wu

**Affiliations:** 1 State Key Laboratory for Crop Genetics and Germplasm Enhancement, Bioinformatics Center, Academy for Advanced Interdisciplinary Studies, Nanjing Agricultural University, Nanjing, China; 2 Jiangbei New Area Biopharmaceutical Public Service Platform Co., Ltd., Nanjing, China; 3 College of Animal Science and Technology, Nanjing Agricultural University, Nanjing, China; 4 Institute of Agricultural Resources and Environment, Jiangsu Academy of Agricultural Sciences, Nanjing, China; 5 Plant Biology Section, School of Integrated Plant Science, Cornell University, Ithaca, USA

**Keywords:** *N*^4^-acetyldeoxycytosine (4acC), 5mC, Histone modification, Gene expression, *Arabidopsis thaliana*

## Abstract

**Background:**

Direct analogs of chemically modified bases that carry important epigenetic information, such as 5-methylcytosine (m5C)/5-methyldeoxycytosine (5mC), 5-hydroxymethylcytosine (hm5C)/5-hydroxymethyldeoxycytosine (5hmC), and *N*^6^-methyladenosine (m6A)/*N*^6^-methyldeoxyadenosine (6mA), are detected in both RNA and DNA, respectively. The modified base *N*^4^-acetylcytosine (ac4C) is well studied in RNAs, but its presence and epigenetic roles in cellular DNA have not been explored.

**Results:**

Here, we demonstrate the existence of *N*^4^-acetyldeoxycytosine (4acC) in genomic DNA of *Arabidopsis* with multiple detection methods. Genome-wide profiling of 4acC modification reveals that 4acC peaks are mostly distributed in euchromatin regions and present in nearly half of the expressed protein-coding genes in *Arabidopsis*. 4acC is mainly located around transcription start sites and positively correlates with gene expression levels. Imbalance of 5mC does not directly affect 4acC modification. We also characterize the associations of 4acC with 5mC and histone modifications that cooperatively regulate gene expression. Moreover, 4acC is also detected in genomic DNA of rice, maize, mouse, and human by mass spectrometry.

**Conclusions:**

Our findings reveal 4acC as a hitherto unknown DNA modification in higher eukaryotes. We identify potential interactions of this mark with other epigenetic marks in gene expression regulation.

**Supplementary Information:**

The online version contains supplementary material available at 10.1186/s13059-021-02578-7.

## Background

Modifications of nucleic acids of genomic DNA (gDNA) naturally exist in cells. More than 17 types of modified nucleosides have been identified in DNA [1], such as 5-methyldeoxycytosine (5mC), 5-hydroxymethyldeoxycytosine (5hmC), 5-carboxydeoxycytosine (5caC), 5-formyldeoxycytosine (5fC), *N*^4^-methyldeoxycytosine (4mC), 5-hydroxymethyldeoxyuridine (5hmU), 5-formyldeoxyuridine (5fU), and *N*^6^-methyldeoxyadenosine (6mA). These modified nucleosides may carry epigenetic information that regulates gene expression in a variety of cellular processes.

Indeed, the two most prevalent modifications, 5mC and 6mA, have been found to carry biological information in both eukaryotes and prokaryotes [2, 3]. 5mC is widely distributed in the genomes of most eukaryotes, including animals, plants, and fungi [4–7]. In plants, 5mC is mostly located in heterochromatin that is enriched with transposable elements (TEs) and other repetitive DNA sequences, inhibits their transposition and transcription, and accordingly is required for gene silencing and genome stability [4–8]. The 5mC modification exists in sequence contexts of CG, CHH, and CHG (H being A or T) and is regulated in each by distinct methyltransferases [9]. In *Arabidopsis*, CHG methylation is enriched in pericentromeric regions because of its preference for TEs [10, 11]. In contrast, CG and CHH methylation, despite being most enriched in the pericentromeric regions, is also observed across the euchromatin regions [8]. A large number of highly transcribed genes are methylated within their coding regions [12–15].

6mA has long been known to be present in prokaryotes and ancient eukaryotes, but its distribution and function among various eukaryotic species have been reported only recently [16–22]. In *Chlamydomonas* and fungi, it is enriched around TSSs (transcription start sites) and appears to mark active genes [18, 21]. In rice, 6mA is depleted from the TSS and is associated with no expression when it marks in the promoter but is associated with gene transcription when it marks in gene bodies [16]. In *Arabidopsis*, 6mA is widespread throughout the genome but denser in pericentromeric heterochromatin regions, and it is positively associated with the gene expression level [17]. In *Drosophila* and zebrafish, 6mA is enriched in repetitive elements and does not seem to be associated with protein-coding genes [20, 22]. In *C. elegans*, 6mA shows a wide distribution across all chromosomes, with no one genomic feature being significantly enriched or depleted [19].

Besides 5mC and 6mA, other modifications of gDNA have not been extensively explored. Acetylation of the *N*^4^ position of cytosine (ac4C) is carried out by the acetyltransferases NAT10 and Kre33 in human and yeast, respectively, and the resulting ac4C is a highly conserved RNA modification of rRNA and tRNA as well as eukaryotic mRNA [23–28]. ac4C was initially discovered in bacterial tRNA [29], and subsequent studies have revealed that ac4C helps to increase the high fidelity of protein translation [30, 31]. Two ac4C modification sites have also been identified on the 18S rRNA of yeast and human cells at helices 34 and 45, which are required for biogenesis of the small ribosomal subunit and translation accuracy [23–25]. In 2018, Arango et al. found that ac4C was present in more than 4000 regions of the human transcriptome using antibody-based enrichment, and they revealed that ac4C promotes mRNA stability and translation efficiency [26]. Tardu et al. showed that ac4C also exists in yeast mRNA through ultra-high-performance liquid chromatography tandem mass spectrometry (UPLC–MS/MS) [32]. The latest nucleotide-resolution method for profiling ac4C sites recapitulates both known sites of ac4C on tRNA and 18S rRNA but has not confirmed the existence of ac4C in eukaryotic mRNA [27]. Because direct analogs of chemically modified bases in RNA and DNA are seen for m5C/5mC, hm5C/5hmC, and m6A/6mA [33–36], we explored the existence and function of *N*^4^-acetyldeoxycytosine (4acC) modification in DNA.

In this study, we applied several methods to demonstrate that 4acC is present in the gDNA of *Arabidopsis thaliana*. We performed 4acC immunoprecipitation followed by sequencing (4acC-IP-seq) to obtain the genome-wide profiling of 4acC. 4acC-IP-seq revealed that 4acC peaks are distributed mostly in euchromatin regions and mark approximately half of the protein-coding genes. 4acC is highly enriched around TSSs and is strongly associated with actively transcribed genes. 4acC and 5mC occupy distinct genomic regions, and the 4acC distribution is not directly altered by changes in 5mC. Genes marked with 4acC display a higher 5mC methylation density in the CG context within gene bodies but lower methylation levels in CHG and CHH contexts compared to non-4acC-marked genes. In addition, more than half of the 4acC peaks are colocalized with active histone modification marks. We observed complex relationships of 4acC with 5mC and histone modifications in the context of gene transcription regulation. Taken together, our findings provide evidence that 4acC is a hitherto unknown modification of gDNA and that 4acC is highly correlated with gene expression in *Arabidopsis*.

## Results

### Characterization of 4acC modification in *Arabidopsis* gDNA

**Fig. 1:**
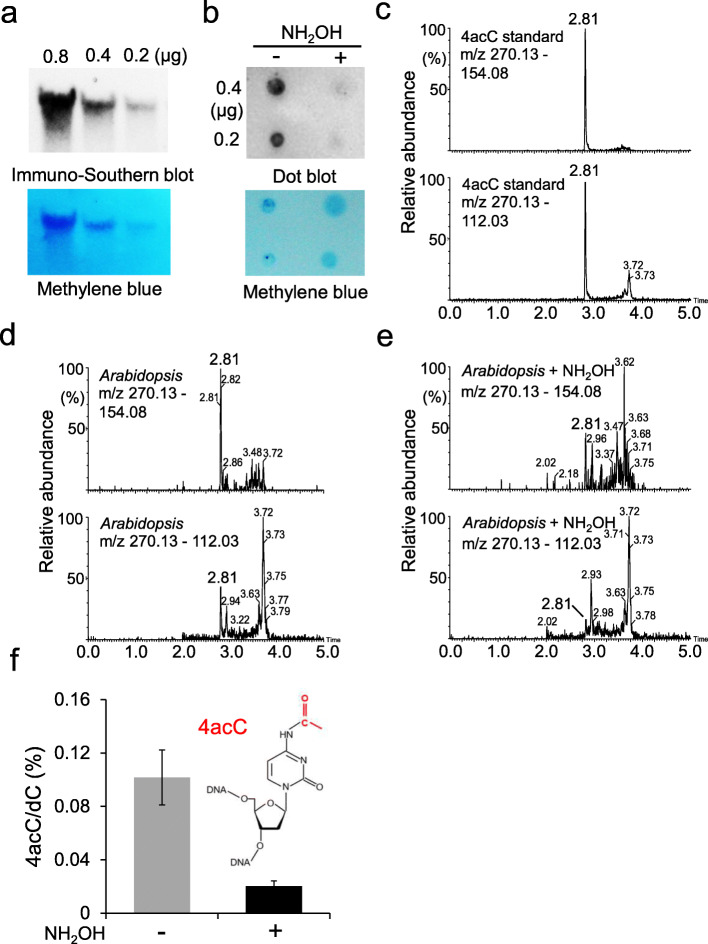
Presence of 4acC in *Arabidopsis* gDNA. **a** Immuno-southern blot of 4acC. A specific anti-4acC antibody was used to detect 4acC signals in gDNA extracted from 3-week-old Col-0 WT plants. The amount of gDNA loaded is indicated. **b** Dot blot showing the effect of hydroxylamine (100 mM, pH 7.0, 65 °C, 2 h) on 4acC in cellular gDNA. The amount of gDNA loaded is indicated. **c−e** UPLC-ESI-MS/MS analysis of 4acC in the 4acC standard (**c**), *Arabidopsis* gDNA (**d**), and *Arabidopsis* gDNA treated with NH_2_OH samples (**e**) with mass transitions of *m/z* 270.13 to 154.08 and *m/z* 270.13 to 112.03 showing peaks at a retention time of approximately 2.81 min. **f** UPLC-ESI-MS/MS quantification of 4acC levels in *Arabidopsis* gDNA samples without or with NH_2_OH treatment. The error bars denote the means ± SDs, *n* = 3 biological replicates

To determine the presence of 4acC in gDNA, we detected 4acC via immunoblot analysis using an antibody that specifically recognizes the ac4C base in mRNA and has been used for genome-wide profiling of ac4C sites in mRNAs [26]. A dot blot assay on a collection of nucleotides revealed that this anti-ac4C antibody recognizes both *N*^4^-acetyl-2’-cytidine (ac4C) and *N*^4^-acetyl-2’-deoxycytidine (4acC) bases but not unmodified 2’-deoxycytidine (dC) or 5-methyl-2’-deoxycytidine (5mC) (Additional file 1: Fig. S1a), indicating its specificity for 4acC. In the immuno-Southern blot assay, *Arabidopsis* gDNA was depleted of RNA by RNase-A and separated from possible residual RNA by gel electrophoresis. The 4acC signals were easily detected on the blots from gels loaded with 0.2 μg of purified gDNA (Fig. 1a). We further treated gDNA with hydroxylamine, which has been shown to deacetylate ac4C in total RNA [37], and found a greatly reduced signals in the treated samples compared to the untreated samples by anti-4acC dot blot (Fig. 1b), supporting the existence of 4acC in DNA.

In addition, we applied a UPLC-electrospray ionization-mass spectrometry (UPLC-ESI-MS/MS) assay to detect and quantify 4acC in the gDNA of *Arabidopsis*. The peaks matching the retention time of approximately 2.81 min of the 4acC standard with mass transitions of *m/z* 270.13 to 154.08 and *m/z* 270.13 to 112.03 were both present in *Arabidopsis* gDNA but not in the mock samples (Fig. 1c–e; Additional file 1: Fig. S1b). The 4acC level in gDNA of 3-week-old Col-0 rosette leaves was 0.1% (4acC/dC) (Fig. 1f). The abundance of 4acC was decreased to 0.02% of that of dC after hydroxylamine treatment (Fig. 1f), further supporting the presence of 4acC in *Arabidopsis* gDNA. Moreover, 4acC was also detected in gDNA samples of rice, maize, mouse, and human (Additional file 1: Fig. S1c-j). These results suggest that 4acC modification is an abundant and general epigenetic mark in higher eukaryotes.

### Genome-wide mapping of 4acC in *Arabidopsis*

To study 4acC distribution and localization in the *Arabidopsis* genome, we performed 4acC-IP (immunoprecipitation)-seq with two independent biological replicates. Similar to the methods for methylated DNA IP (MeDIP )[38] and 6mA-IP-seq [18], which have been widely used to enrich 5mC- and 6mA-containing DNA fragments, we applied the 4acC-specific antibody described above to enrich the 4acC-containing DNA fragments. The gDNA from *Arabidopsis* was fragmented into 200–400 base pairs, end-repaired, and 3′-adenylated before being ligated with a library-constructing adapter. DNA fragments containing 4acC, after being denatured, were immunoprecipitated using the anti-4acC antibody for construction of a high-throughput sequencing library. The input library, not enriched for 4acC, was made from adaptor-ligated DNAs before IP. The obtained sequencing reads were mapped to the *Arabidopsis* genome TAIR10, and the unique reads were used to identify 4acC sites with MACS2 [39] with the cutoff of a false discovery rate (FDR) < 0.05.

**Fig. 2:**
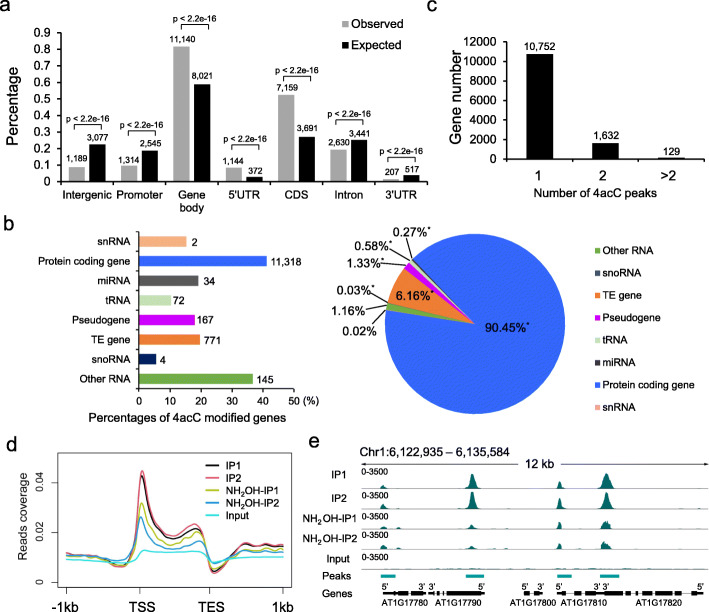
Genome-wide mapping and distribution of 4acC in *Arabidopsis*. **a** Distributions (expressed as percentages) of 4acC peaks found among intergenic regions, promoters (within 1 kb upstream of the TSS), and gene bodies. Gene bodies were further divided into 5′ and 3′ UTRs, exons (excluding 5′ and 3′ UTRs), and introns. The *p* values were calculated by Fisher’s exact test (*p* < 2.2 × 10^−16^). **b** Distributions of 4acC modifications in different gene categories. The left panel shows the numbers and percentages of 4acC-modified genes in different gene categories. The right panel shows the distributions (as percentages) among different gene categories for the 4acC-acetylated genes. The asterisks in the pie chart indicate significant differences between the observed versus expected distributions of 4acC peaks (Fisher’s exact test, *p* < 10^−5^). miRNA, microRNA; tRNA, transfer RNA; snRNA, small nuclear RNA; snoRNA, small nucleolar RNA; TE, transposable element gene. **c** Number of protein-coding genes possessing 1, 2, or > 2 4acC peaks in their gene bodies. **d** 4acC profiles in gene regions. The 1 kb upstream and downstream flanking coding regions were aligned for all genes. TSS, transcription start site; TES, transcription end site. **e** Representative profiles of 4acC-IP and input in the region of chr1:6,122,935-6,135,584

A total of 24–47 million raw reads were generated for each library, and over 90% of these reads were aligned to the *Arabidopsis* genome (Additional file 2: Table S1), indicating high mapping quality. High Pearson correlation coefficients (*R* = 0.96) were observed between the two biological replicates (Additional file 1: Fig. S2a). After peak calling, we identified 14,212 and 15,297 high-confidence 4acC peaks (Additional file 1: Fig. S2b; Additional file 3: Table S2). Among them, over 95% of the peaks mutually occurred in the two replicate samples (Additional file 1: Fig. S2b), indicating the high reproducibility of the results. To explore the epigenetic roles of 4acC, we investigated the 4acC distribution in *Arabidopsis* genomic regions, including intergenic regions, promoters (within 1 kb upstream of the TSS), gene bodies, and their subregions. We found that 82% of 4acC peaks were located on gene bodies, and half of them were located in exons (Fig. 2a). Protein-coding genes composed the largest group of 4acC-acetylated genes (Fig. 2b, right panel), and 41% of the protein-coding genes in the genome contained 4acC modifications (Fig. 2b, left panel). 4acC was also detected in other types of genes, such as pseudogenes, TE genes, and noncoding RNA genes (Fig. 2b, left panel). Most protein-coding genes contained one 4acC peak in the gene body (Fig. 2c). To further analyze the distribution pattern of 4acC in genes, we plotted 4acC-IP and input reads throughout coding regions and 1 kb upstream and downstream for all genes. The 4acC position genome-wide was enriched near the TSS in *Arabidopsis* (Fig. 2d). This TSS-enriched pattern was also observed in maize (Additional file 1: Fig. S2c), implying that 4acC may show a similar distribution pattern in plants. A representative feature of the 4acC distribution is shown in Fig. 2e. To verify that these peaks indeed represented 4acC modification, we treated DNAs with hydroxylamine to partially remove the 4acC modification. 4acC IP-seq analysis of the hydroxylamine-treated samples revealed a substantial reduction, but not elimination, of almost all peaks in the treated samples compared to the nontreated samples (Fig. 2d, e), suggesting that the peaks were indeed specific to 4acC modification. Thus, 4acC modification is region-specific and is highly enriched around the TSSs of protein-coding genes.

### Correlation between 4acC modification and transcription

**Fig. 3:**
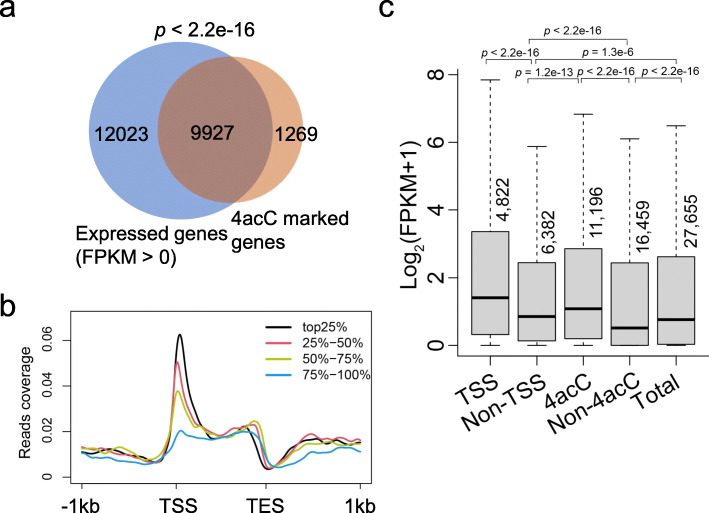
Correlation of 4acC with gene expression. **a** Venn diagram showing the overlaps between expressed genes and 4acC-marked genes. An expressed gene was defined by having an FPKM > 0. FPKM stands for the fragments per kilobase of exon per million fragments mapped from RNA-seq data. The *p* values were calculated by Fisher’s exact test. **b** Distributions of 4acC peaks over protein-coding gene regions among groups of genes with high to low (top 25%, 25–50%, 50–75%, and 75–100% based on FPKM) expression levels. **c** Box plot of gene expression levels (based on FPKM values) for genes with or without 4acC peaks (4acC and non-4acC) as well as genes with 4acC peaks around the TSS (TSS) or away from the TSS (non-TSS). The *p* values were calculated for significant differences between two groups by the Mann–Whitney *U* test

The high enrichment of 4acC peaks around the TSSs in *Arabidopsis* genes prompted us to investigate the relationship of 4acC with gene expression. To this end, we performed RNA-seq with two biological replicates to analyze individual gene expression, which showed high Pearson correlation coefficients (*R* = 0.99) (Additional file 1: Fig. S3a). According to the RNA-seq data, 21,950 and 12,615 genes were detected with FPKM > 0 and FPKM > 1 (FPKM represents the fragments per kilobase of exon per million fragments), respectively (Fig. 3a; Additional file 1: Fig. S3b). Among these expressed genes with FPKM values > 0 or 1, over 45% or 46% of them contained 4acC modification, respectively. In addition, 89% or 51% of the 4acC-marked genes were expressed with FPKM values > 0 or 1, respectively. We further categorized the Gene Ontology (GO) terms of the expressed genes (FPKM > 0) with or without 4acC modification. The use of agriGO v2.0 [40] revealed 192 terms enriched for 4acC-containing genes (Additional file 4: Table S3), but only 24 terms enriched for non-4acC-containing genes (Additional file 5: Table S4). This suggests that 4acC-modified expressed genes are involved in more diverse biological functions than non-4acC expressed genes.

To explore the association of 4acC with gene expression, all protein-coding genes in the genome were divided into four groups according to their expression level: top 25%, 25–50%, 50–75%, and 75–100%. Plotting of 4acC abundance in these four groups revealed that strongly expressed genes had a higher occupancy of 4acC around TSS regions than weakly expressed genes (Fig. 3b). At a genome-wide level, genes containing 4acC were expressed at significantly higher levels than genes without 4acC modification, and furthermore, genes with 4acC peaks around the TSS (in a 250-bp window centered on the TSS) had higher expression than genes with non-TSS-region modification (Fig. 3c). Therefore, 4acC modification, especially at the TSS region, is strongly correlated with gene expression.

### Relationships between 4acC and 5mC DNA modification

**Fig. 4:**
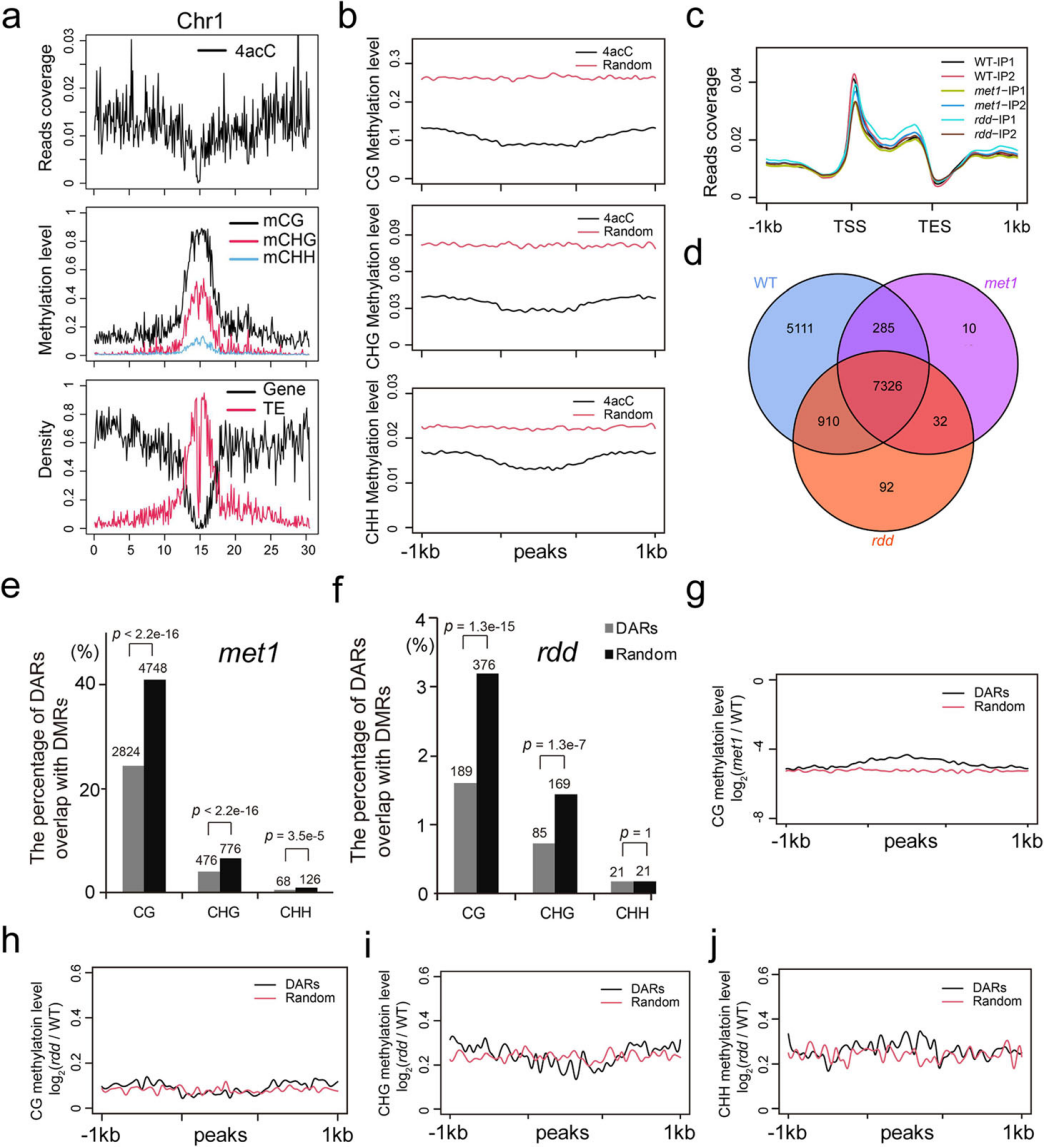
Relationships between 4acC and 5mC in the *Arabidopsis* genome. **a** 4acC distribution in *Arabidopsis* chromosome 1. The top panel shows the average 4acC levels per 100-kb bin. The middle panel shows the average 5mC levels at CG, CHG, and CHH sites, and the bottom panel shows the densities of genes and TEs. **b** The average levels of 5mC (CG, CHG, and CHH) in 4acC peaks with 1 kb upstream and downstream flanking regions and random regions. **c** 4acC profiles of *met1* and *rdd* mutants in gene regions. The 1 kb upstream and downstream flanking coding regions were aligned for all genes. TSS, transcription start site; TES, transcription end site. **d** Overlaps of 4acC peaks among WT, *met1* mutant and *rdd* mutant plants. **e, f** Percentages of DARs that overlapped with DMRs in *met1* (**e**) and *rdd* (**f**) mutants. The *p* values in **e** and **f** were calculated for significant differences between two groups by Fisher’s exact test. **g** Fold changes in mCG levels in DARs and randomly selected regions between the *met1* mutant and WT plants. **h–j** Fold changes in mCG (**h**), mCHG (**i**), and mCHH (**j**) levels in DARs and randomly selected regions between the *rdd* mutant and WT plants

Given that 4acC and 5mC are both abundant DNA modifications present in the *Arabidopsis* genome, we investigated whether these two modifications interact with each other. First, the distribution of 4acC was examined along the chromosomes. In contrast to 5mC, which was enriched in pericentromeric heterochromatin, 4acC peaks were mostly located in euchromatin regions (Fig. 4a; Additional file 1: Fig. S4a). Thus, 4acC and 5mC appear to occupy distinct regions of the genome. Second, the distribution and modification levels of 5mC in 4acC peaks and their 1 kb upstream and downstream regions were analyzed. 4acC-enriched regions contained lower mCG, mCHG, and mCHH levels than randomly selected regions (Fig. 4b), further supporting the idea that 4acC and 5mC are distributed in different positions.

To examine whether an imbalance in 5mC affects 4acC modification, we profiled genome-wide 4acC modifications in two DNA methylation mutants, *met1* and *ros1dml2dml3* (*rdd*). Mutation of the methyltransferase MET1 leads to the elimination of CG methylation throughout the genome [41, 42], while hypermethylation occurs in all three cytosine contexts in the DNA demethylase mutant *rdd* [42–44]. The TSS-enriched distribution pattern of 4acC found in the Col-0 wild-type (WT) was also observed in *met1* and *rdd* mutants (Fig. 4c). However, the global 4acC abundances were decreased in both *met1* and *rdd* mutants compared to WT plants (Additional file 1: Fig. S4b). After peak calling, 8024 and 8876 4acC peaks were identified in the *met1* and *rdd* mutants, respectively, and 98% of them overlapped with peaks identified in the WT plants (Fig. 4d; Additional file 1: Fig. S4c, d; Additional file 6: Table S5 and 7: Table S6). Differentially acetylated regions (DARs) and differentially methylated regions (DMRs) were identified between the mutants and WT plants (Additional file 1: Fig. S4e, f; Additional file 8: Table S7; Additional file 9: Table S8; Additional file 10: Table S9). The DARs in both *met1* and *rdd* mutants showed significantly less overlap with DMRs than random regions, implying that DARs were not associated with DMRs (Fig. 4e, f). In addition, DARs in the *met1* mutant displayed slightly lower fold changes in mCG levels than randomly selected regions (Fig. 4 g) because of lower DNA methylation in the 4acC-enriched regions. Furthermore, DARs in the *rdd* mutant had fold changes in mCG, mCHG, and mCHH levels similar to those of randomly selected regions (Fig. 4h–j). Therefore, the changes in 4acC in *met1* and *rdd* mutants are not directly associated with alterations in 5mC modification. The global reduction in 4acC levels may be caused by the combined effects of alterations of multiple epigenetic marks or writers or erasers of 4acC in *met1* and *rdd* mutants.

To investigate the alterations in 4acC levels affecting gene expression, we analyzed the overlaps between differentially expressed genes (DEGs) and unique differentially acetylated genes (uDAGs) in the *met1* mutant compared to the WT. The uDAGs were defined as generic regions or 1-kb regions upstream of DAGs that did not overlap with differentially methylated regions between *met1* and WT plants. We employed RNA-seq data of the *met1* mutant from a previous study [42] and found that 45% of uDAGs showed significantly differential expression between *met1* and WT plants. In addition, 36% of DEGs overlapped with uDAGs in the *met1* mutant (Additional file 1: Fig. S4g), suggesting that 4acC plays a role in gene expression regulation.

### Cooperative interactions of 4acC and 5mC modifications in gene expression regulation

**Fig. 5:**
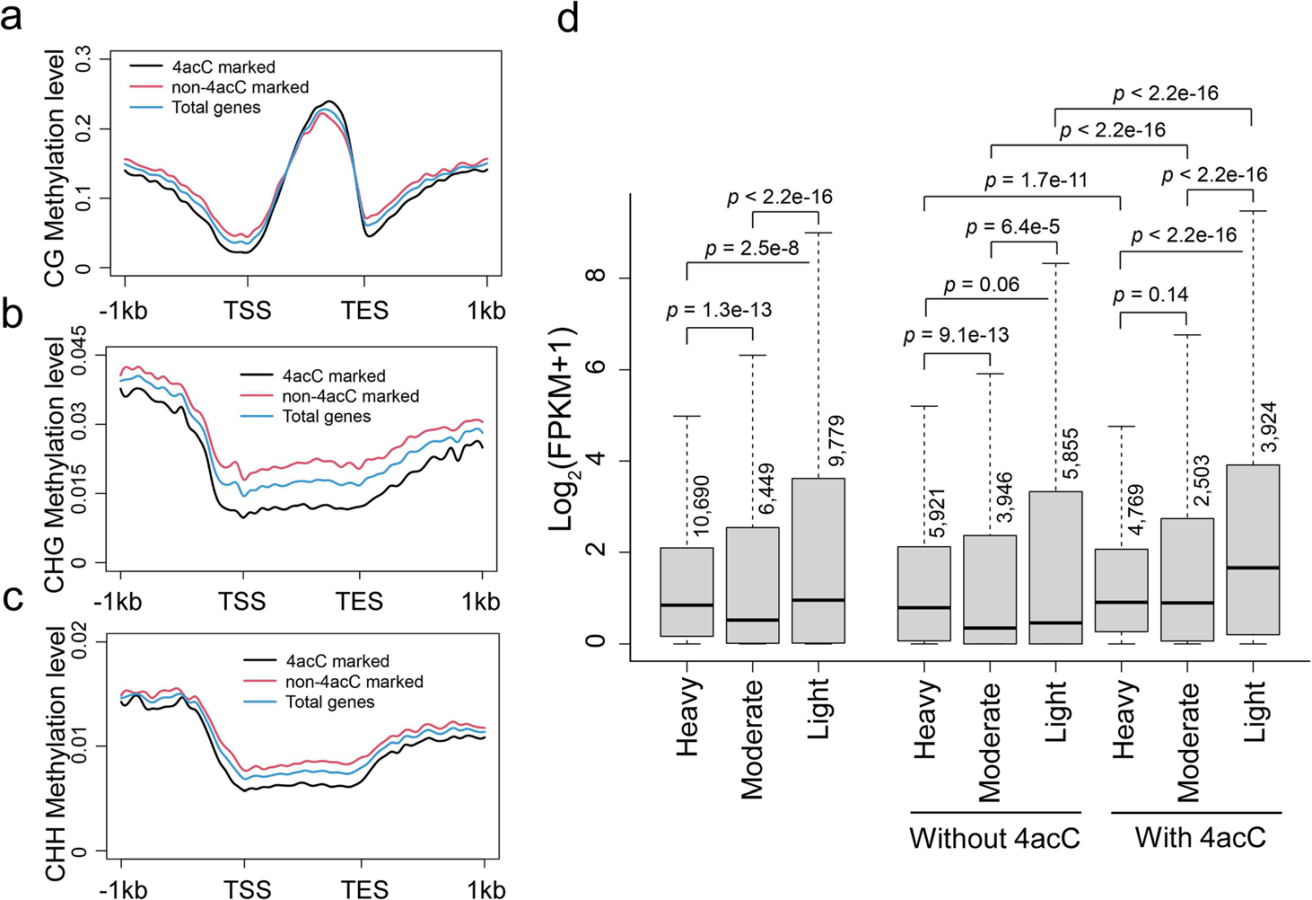
Cooperative interactions of 4acC and 5mC modifications to affect gene expression. **a**–**c** Genome-wide average levels of mCG (**a**), mCHG (**b**), and mCHH (**c**) in total genes with or without 4acC across the gene region. **d** Box plots of the expression levels of genes marked by heavy, moderate, and light levels of mCG only or mCG together with 4acC in gene body regions. The mCG levels were calculated according to the ratio of mCG to all cytosines located at each position. Genes with average ratios of ≥ 0.1, 0.01 to 0.1, and ≤ 0.1 were defined as heavily, moderately, and lightly methylated, respectively. The gene number in each modification group is indicated. The *p* values were calculated for significant differences between two groups by the Mann–Whitney *U* test

Since genes methylated by 5mC in the gene body have also been found to have high expression [12–15], we compared the distributions of 4acC and 5mC in protein-coding genes and investigated their cooperative effects on gene expression. The densities of CG, CHG, and CHH methylation were calculated over each gene for 4acC-marked and non-4acC-marked genes, respectively. Notably, in contrast to the finding that 4acC-enriched regions contain lower CG, CHG, and CHH methylation levels than random regions (Fig. 4b), the 4acC-marked genes displayed higher mCG methylation levels within gene bodies (Fig. 5a) but lower levels of mCHG and mCHH contexts than the non-4acC-marked genes (Fig. 5b, c). Almost all genes contained mCG (Additional file 1: Fig. S5), and genes with heavy, moderate, and light mCG modifications exhibited medium, lower, and higher levels of gene expression (Fig. 5d). Therefore, the extent of mCG was not significantly associated with gene expression. Interestingly, an association between mCG levels and gene expression was observed when genes with or without 4acC were separately considered. For genes without 4acC, high-mCG genes had higher expression than moderate- or low-mCG genes, while for those with 4acC, low-mCG genes had higher expression than genes with high or moderate mCG (Fig. 5d). Therefore, for genes with moderate or low mCG, having 4acC was strongly associated with increased gene expression. Among the different 4acC and mCG combinations, low mCG with 4acC was associated with the highest gene expression, while moderate or low mCG without 4acC was associated with the lowest expression (Fig. 5d). These data suggest that 4acC has a positive effect on gene expression and that its effect is most drastic in genes with low mCG. These data also offer an explanation for the loose association between mCG levels and gene expression levels, as 4acC is another important contributor to gene expression levels.

### Colocalization and interaction between 4acC and histone modification marks

**Fig. 6:**
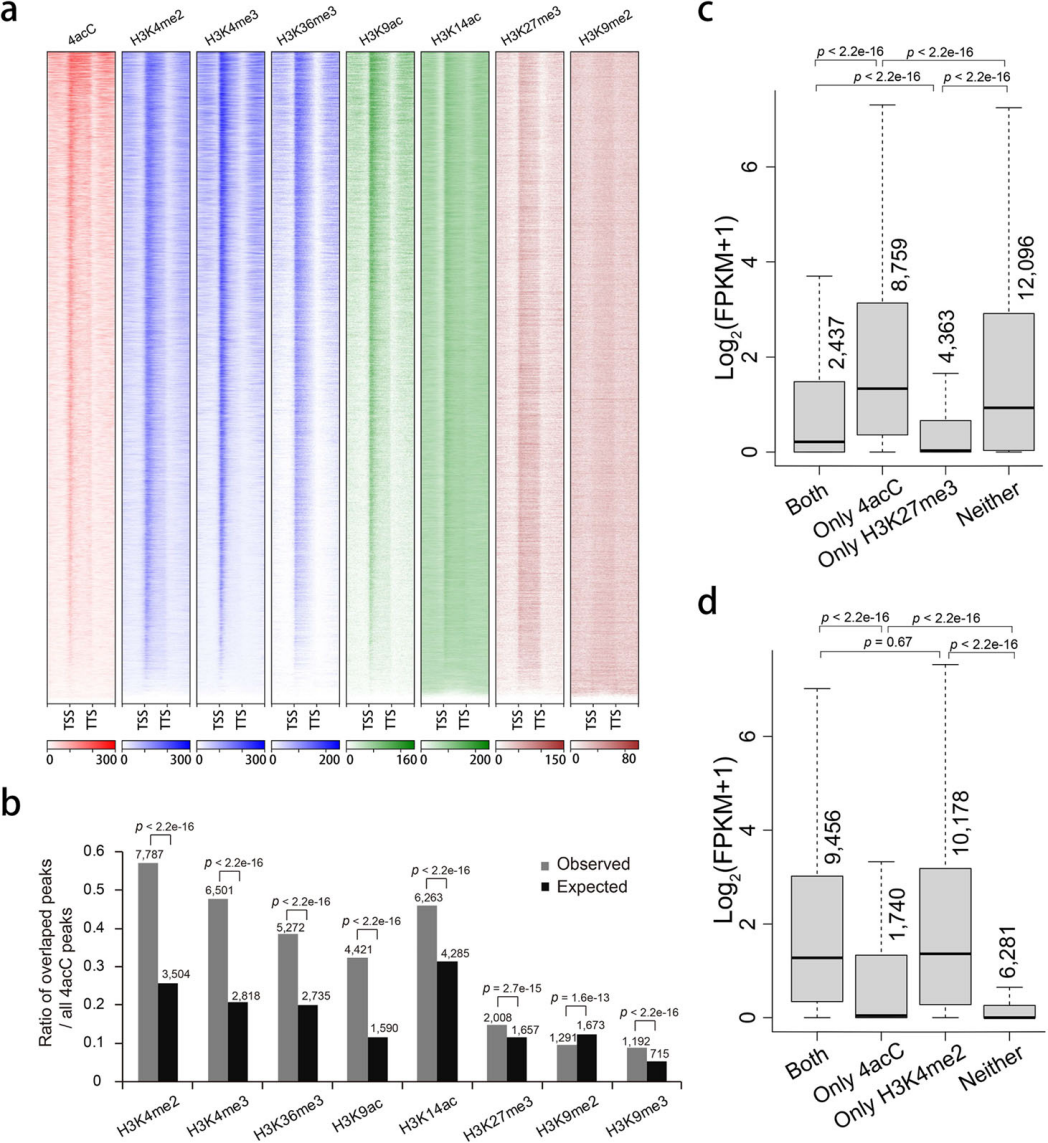
Colocalization and interactions between 4acC and histone modification marks. **a** Heatmap of 4acC and seven histone modification marks in genes with 1 kb upstream and downstream flanking regions. **b** Ratios (expressed as percentages) of peaks with histone modification marks for genes containing 4acC peaks. The peak number of each group is shown. Significant differences between the observed ratio and the expected ratio due to chance were analyzed by Fisher’s exact test, and the *p* values are shown. **c**, **d** Box plot of expression levels for genes marked or not marked by H3K27me3 (**c**) or H3K4me2 (**d**) in combination with 4acC or a lack of 4acC in gene body regions. The *p* values were calculated for significant differences between two groups by the Mann–Whitney *U* test

The features of 4acC modification, high enrichment around the TSS and positive correlation with gene expression, prompted us to examine the colocalization of 4acC with active chromatin marks. We observed that 4acC colocalized significantly with active modification marks, including H3K4 di/trimethylation (H3K4me2/3), H3K36 trimethylation (H3K36me3), H3K9 acetylation (H3K9ac), and H3K14 acetylation (H3K14ac) (Fig. 6a, b). In total, 32 to 57% of the 4acC peaks showed overlaps with active modification marks, significantly higher than the percentage of overlaps that would occur by random chance (Fig. 6b). Surprisingly, 15% of 4acC peaks displayed overlaps with the repression mark H3K27 trimethylation (H3K27me3), which was also significantly higher than the percentage of overlaps that would occur by chance (Fig. 6b). The overlaps of 4acC peaks with another repression mark, H3K9 dimethylation (H3K9me2), were significantly lower than expected (Fig. 6b). In general, the percentages of overlaps of 4acC peaks with repressive histone modification marks were lower than those of overlaps with active modification marks. Therefore, 4acC shows high colocalization with active histone modification marks.

We subsequently analyzed the combined impact of histone modification and 4acC on gene expression levels. A positive effect of 4acC on gene expression was observed when genes with or without repressive histone modification marks were separately analyzed. As observed before, genes with H3K27me3 had lower expression than genes without H3K27me3 modification irrespective of the 4acC modification (both versus 4acC, H3K27me3 versus neither, as in Fig. 6c). Strikingly, having 4acC was associated with increased expression within each of the two groups of genes with or without H3K27me3 (both versus H3K27me3, 4acC versus neither, as in Fig. 6c), and the effect was larger in genes without H3K27me3 (Fig. 6c). A similar pattern was observed for one other repressive histone modification mark, H3K9me2 (Additional file 1: Fig. S6a).

The impact of 4acC on gene expression was also investigated when genes with or without an active histone mark were separately considered. Irrespective of 4acC modification, H3K4me2-marked genes had higher expression than non-H3K4me2-modified genes (Fig. 6d). Within the group of genes without H3K4me2 modification, the genes marked by 4acC had significantly higher expression than those without 4acC (Fig. 6d). Similar patterns were also observed for other active histone modification marks, such as H3K4me3, H3K36me3, H3K14ac, H3K9ac, and H3K9me3 (Additional file 1: Fig. S6d-f). However, 4acC displayed distinct impacts within a group of genes with active histone modification marks. Genes with 4acC had similar expression to those without 4acC within the group of H3K4me2-marked genes (Fig. 6d) but displayed higher expression than those without 4acC within the group of genes with H3K14ac (Additional file 1: Fig. S6c) and lower expression within the other four groups of genes with H3K4me3, H3K36me3, H3K9ac, and H3K9me3 (Additional file 1: Fig. S6a, b, d). Therefore, 4acC is associated with high expression under specific histone modification statuses, and its impact is most significant on genes without active or with repressive histone marks.

### Colocalization of 4acC with DNase I hypersensitive sites (DHs) and transcription factor (TF) binding sites

**Fig. 7:**
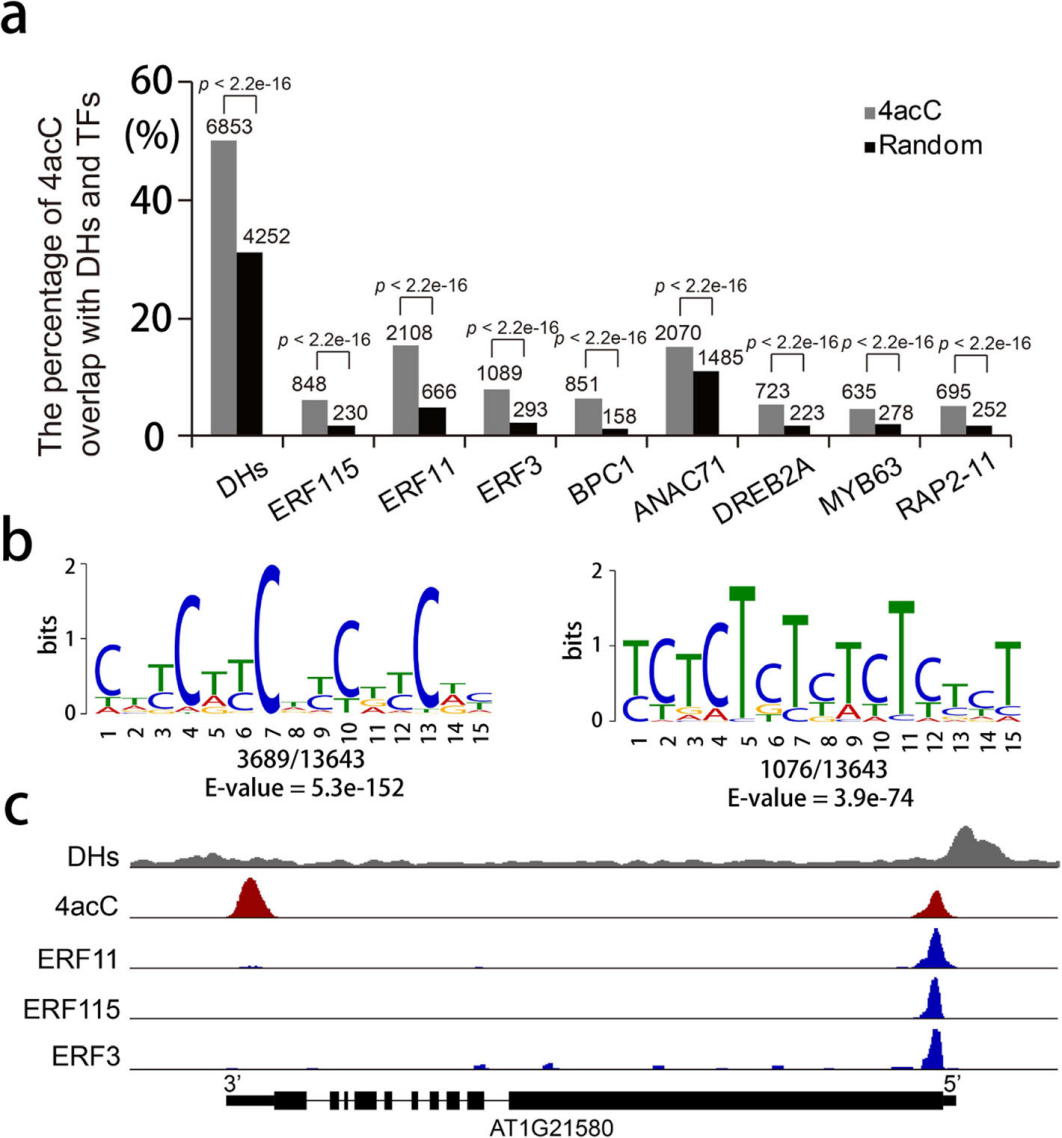
Overlaps of 4acC with DHs and TF binding sites. **a** The percentages of 4acC overlap with DHs and the binding sites of TFs, including ERF3, ERF11, ERF115, BPC1, ANAC71, DREB2A, MYB63, and RAP2-11. The *p* values were calculated for significant differences by Fisher’s exact test. **b** Consensus motifs associated with 4acC peaks. Shown under the logo of each motif is the number of occurrences/the total number of 4acC-containing motifs and the corresponding E-value generated by MEME-ChIP. **c** Representative features of 4acC colocalized with DHs and the binding sites of ERF3, ERF11, and ERF115 at the 5′ end of the *AT1G21580* gene

Furthermore, we investigated the overlaps between 4acC and DHs and found that nearly 50% of 4acC peak regions overlapped with DHs [45] (Fig. 7a), indicating that a considerable proportion of 4acC-modified regions may contain regulatory DNA elements. Therefore, we carried out an unbiased search using MEME-ChIP [46] for consensus motifs in 4acC peak regions. Dozens of motifs were significantly enriched, such as CDYCDYCDYCDYCDY (D represents A, G and T; Y represents C and T; E-value = 5.3 × 10^−152^) and YCTCTCTYTCTYYYT (E-value = 3.9 × 10^−74^), which are known or similar motifs of many classes of TFs (Fig. 7b; Additional file 11: Table S10). Indeed, analysis of DAP-seq data [47] for these TFs, including ERF3, ERF11, ERF115, BPC1, ANAC71, DREB2A, MYB63, and RAP2-11, showed significant overlaps with 4acC-modified regions (Fig. 7a). A representative scene is shown in Fig. 7c. Therefore, 4acC may facilitate the binding of TFs to regulate gene expression.

## Discussion

In this study, we identified 4acC as a hitherto unknown form of DNA modification in the genomes of several model higher eukaryotes, including *Arabidopsis*, rice, maize, mouse and human. In *Arabidopsis*, 4acC peaks are distributed mostly in euchromatin regions and are mainly present around the TSS regions of protein-coding genes. In the more complex genome of maize, 85% of which is constituted by transposons, 4acC also shows high enrichment around TSSs, implying that 4acC may share similar epigenetic modification systems in different species. Furthermore, 4acC is positively correlated with the gene expression level. Whether 4acC modification is a general epigenetic mark pertaining to gene transcription in other organisms remains to be explored. For instance, we also detected 4acC in two mammalian genomes by mass spectrometry. More extensive experiments such as 4acC-IP-seq in mammals would not only further validate the existence of 4acC as a popular epigenetic marks in eukaryotes, but also shed light on the evolutional conservation and divergence of 4acC biological function.

Specific chromatin states are associated with the patterns of gene activity and gene silencing. In mammals and plants, restrained heterochromatin accumulates with 5mC hypermethylation of DNA and with repressive modification of histones, such as the methylation of H3K9. Here, we show that 4acC is distributed mostly in euchromatin regions and is strongly associated with expressed genes. Several observations indicate that 4acC DNA acetylation may act as an epigenetic mark for euchromatin. Strongly expressed genes display a higher 4acC occupancy around the TSS regions than weakly expressed genes. In addition, 4acC-marked genes with low mCG or active histone modifications show high levels of expression. 4acC and 5mC are both abundant DNA modifications on cytosine, but they occupy distinct regions of the *Arabidopsis* genome. Even though the global 4acC level was decreased in the *met1* and *rdd* mutants, the DARs were not correlated with the changes in 5mC. The reductions in 4acC levels may have been caused by the combined effects of alterations in multiple epigenetic marks in *met1* and *rdd* mutants. 4acC-marked genes have higher CG methylation but lower CHG and CHH methylation in gene body regions than non-4acC-marked genes. The different distribution patterns of mC among the CG, CHG, and CHH contexts in 4acC-marked genes imply a complex regulatory mechanism that coordinates the two DNA modifications in *Arabidopsis*. Previous studies have found that genes with 5mC methylation in the coding regions are likely expressed at higher levels than genes without 5mC [7]. Here, we found that genes with mCG in the gene body have higher expression only when they are also modified by 4acC than those without 4acC. These findings suggest cooperative interactions between 4acC and 5mC to affect gene transcription. Both 4acC and 5mC are prevalent chemical modifications deposited on cytosine, and their interactions in epigenetic roles and biological functions need further investigation.

A complex interplay between DNA methylation and histone methylation has been revealed in previous studies. For instance, 5mC methylation has been found to control histone H3K9 methylation [48], and H3K9me2 and 5mC methylation mutually enhance each other in *Arabidopsis* [49]. Here, we show that a large number of 4acC peaks colocalize with active histone modification marks, such as H3K4me2/3, H3K36me3, and H3K14ac. Genes marked by active histone modifications, with or without 4acC, displayed higher expression than genes not marked by active histone modifications, indicating a dominant effect of active histone modification over 4acC on gene expression. Interestingly, 4acC-marked genes had higher expression than non-4acC-marked genes when they were not marked by active histone modification marks or were marked by repressive histone modifications. These data strongly suggest that 4acC is a critical player in positively regulating gene expression when there is a lack of active histone modification marks. One hypothesis is that 4acC acts as a DNA mark that enhances transcription. It may recruit active histone marks to elevate expression even further. How 4acC participates in gene expression regulation and its associations with other epigenetic marks will be of great interest for further investigation. We suggest two efforts in the future to facilitate the related studies. One is identification of the writer, eraser, and reader of 4acC. The other is synthesis of DNA containing 4acC in vitro, which is necessary for further biochemical characterization (e.g., restriction enzyme digestion and chromatography). Nevertheless, it has not yet been settled due to lack of dCTP labeled with 4acC modification.

Taken together, the findings of this study uncover 4acC as an abundant DNA modification in the *Arabidopsis* genome. It is enriched around transcription start sites and preferentially marks actively transcribed protein-coding genes. The genome distribution and modification motifs of 4acC are distinct from those of 5mC, and the two DNA modifications combinatorically contribute to gene expression. 4acC has a large overlap with active histone modification marks, and 4acC is associated with increased expression of genes without active histone marks. The identification of 4acC DNA modification with a gene expression impact will open a new area of epigenetic research.

## Conclusions

In summary, we reveal 4acC as a novel and abundant DNA modification in the genomes of several plant species. In *Arabidopsis*, 4acC is located primarily around the TSSs of protein-coding genes among euchromatin regions and positively correlates with gene expression level. We observed potential interactions of this mark with other epigenetic marks in gene expression regulation. However, several additional questions need to be investigated, particularly what acetyltransferase mediates 4acC modification, the roles of 4acC in gene expression regulation, and the crosstalks with other epigenetic marks. Our findings extend the catalog of DNA modifications regulating gene expression.

## Methods

### Plant materials and growing conditions

*Arabidopsis thaliana* Col-0 WT plants, first-generation homozygous *met1* (*met1-3*) mutants, and *rdd* (*ros1-4 dml2-2 dml3-2*) mutants were grown in a growth chamber at 22 °C under long-day conditions (16 h light/8 h dark) with a light intensity of 100 μmol m^−2^ s−^1^ for 3 weeks. Rice (*Oryza sativa*, *Nipponbare*) and maize (*Zea mays*, *B73*) were grown in a growth chamber at 28 °C under long-day conditions (16 h light/8 h dark) with a light intensity of 200 μmol m^−2^ s^−1^ for 2 weeks.

### Extraction of gDNA from plants and animals

gDNA of *Arabidopsis*, rice and maize seedlings was extracted by using a DNAsecure Plant Kit (TIANGEN, Cat. DP320-03) according to its procedures. Twenty-five milligrams of mouse liver tissue was ground in liquid nitrogen to extract high-purity gDNA using a TaKaRa MiniBEST Universal Genomic DNA Extraction Kit (TaKaRa, Cat. 9765) according to the product manual. Human 293 T cells (5.0 × 10^6^) were cultured in Dulbecco’s modified Eagle’s medium (Thermo Fisher Scientific, Cat. A4192101) supplemented with 10% fetal bovine serum (Thermo Fisher Scientific, Cat. A3840001) and 1% penicillin-streptomycin (Thermo Fisher Scientific, Cat. 15140122) in a humidified incubator at 5% CO_2_ and 37 °C, and then high-purity genomic DNA was extracted using a TaKaRa MiniBEST Universal Genomic DNA Extraction Kit (TaKaRa, Cat. 9765) according to the product manual.

### Dot blot assay of the specificity of the anti-ac4C antibody for 4acC modifications

Different amounts of the pure nucleosides ac4C (Carbosynth, NA05753), 4acC (Adamas, 48652), 5mC (Sigma, M4254), and dC (Sigma, D3897) or DNA samples that were denatured at 95 °C for 5 min were spotted on a Hybond-N+ membrane (Amersham). After UV crosslinking, the membrane was blocked with 5% nonfat milk in PBST for 1 h at room temperature and then incubated with an anti-ac4C antibody (1:1000; Abcam, ab252215) overnight at 4 °C. After washing with TBST, the membrane was incubated with a horseradish peroxidase (HRP)-conjugated anti-rabbit IgG secondary antibody (1:10,000; Abcam, ab6721) for 30 min at room temperature. The membrane was then washed with TBST and treated with High-sig ECL Western Blotting Substrate (Tanon, 180-501). Finally, the signal was detected with a Tanon-5200 Multi instrument. For hydroxylamine treatment, gDNA was first denatured at 95 °C for 5 min, immediately chilled on ice, and then incubated with 100 mM hydroxylamine at 65 °C for 2 h.

### Immuno-Southern blot detection of 4acC in gDNA

gDNA was treated with RNase A to digest RNA. After purification, different amounts of DNA samples were loaded in a 0.8% agarose gel containing 1 mg/L ethidium bromide. The electrophoresis gel was immersed in 0.25 mol/L HCl solution for 5 min for depurination and then moved to an alkaline solution (1.5 M NaCl, 0.5 M NaOH) for 30 min to break the DNA into shorter single-stranded DNA fragments. Finally, it was soaked in neutralization buffer (1 M Tris-HCl pH 7.4, 1.5 M NaCl) to neutralize the gel. The DNA was transferred onto a Hybond-N+ membrane (Amersham) by capillary transfer using 10× SSC buffer (1.5 M NaCl, 0.15 M Nacitrate). The membrane was rinsed with 2× SSC buffer and crosslinked for 5 min (HL-2000 HybriLinker). The rest of the procedures were the same as those for the dot blot assay. For full Immuno-Southern blots, see Additional file 1: Fig S7.

### UPLC-ESI-MS/MS analysis of 4acC in genomic DNA

Two micrograms of purified genomic DNA was denatured at 95 °C for 10 min and subsequently chilled on ice. Then, the DNA samples were digested with 2 U of DNase I (Roche, 04716728001) for 2 h at 37 °C followed by 4 U of calf intestinal alkaline phosphatase (NEB, M0290V) and 0.008 U of phosphodiesterase I (Sigma, P3243) at 37 °C overnight. After the enzymes were removed by ultrafiltration, and the digested DNA was subjected to UPLC-ESI-MS/MS analysis. UPLC-ESI-MS/MS analysis was performed on a Waters Xevo TQ-S micro mass spectrometer (Waters, Milford, MA, USA) with an electrospray ionization source (ESI) and an Acquity UPLC-I-Class™ System (Waters, Milford, MA, USA). Data acquisition and processing were performed using Masslynx software (version 4.1, Waters, Manchester, UK). UPLC separation was performed on a reverse-phase BEH-C18 column (2.1 × 50 mm, 1.7 μm; Waters) with a flow rate of 0.3 mL/min at 35 °C. FA in water (0.1%, v/v, buffer A) and methanol (buffer B) were employed as the mobile phase. The gradient was as follows: 0 min 98% A, 2% B; 0 to 0.5 min 98% A, 2% B; 0.5 to 2 min 90% A, 10% B; 2 to 3 min 10% A, 90% B; 3 to 3.5 min 98% A, 2% B; and 3.5 to 5 min 98% A, 2% B. Mass spectrometry detection was operated under positive electrospray ionization mode. The 4acC retention time was approximately 2.81 min. The mass transitions were monitored as follows: *m/z* 270.13-154.08 and *m/z* 270.13-112.03 for 4acC and *m/z* 228.1-112.1 for dC.

### 4acC-IP-Seq

Five micrograms of purified gDNA was sonicated into 200−400-bp fragments with a Biorupter UCD-600. Then, end repair, 3′-adenylation, and adapter ligation were performed. The ligated and purified DNA was denatured at 95 °C for 10 min and immediately chilled on ice. Ten percent of the denatured DNA was saved as input. The remaining DNA was incubated with 3 μg of anti-ac4C antibody (Abcam, ab252215) at 4 °C for 6 h in 1× IP buffer (10 mM Tris-HCl pH 7.4, 150 mM NaCl, 0.1% IGEPAL CA-630). In addition, Dynabeads Protein A (Invitrogen, 10001D) were washed twice with 1× IP buffer and preblocked in 0.5 ml of 1× IP buffer with 20 mg/ml BSA for 2 h at 4 °C. After two washes with 1× IP buffer, the preblocked beads were coincubated with the DNA-antibody mixture with rotation overnight at 4 °C. The Dynabeads were collected with a magnet and washed 4 times with 1× IP buffer at room temperature. The beads were suspended in 250 μl of proteinase K digestion buffer with 50 μg of proteinase K and digested at 50 °C for 1.5 h. The DNA was purified by phenol/chloroform extraction followed by ethanol precipitation and suspended in 10 μl of ddH_2_O. An NEB Next Ultra II DNA Library Prep Kit for Illumina (NEB, E7645S) was used to construct the library with PCR amplification for 15 cycles. The DNA was purified with AMPure beads and finally suspended in 18 μl of ddH_2_O to yield the sequencing library.

Two biological replicates were sequenced using the Illumina NovaSeq6000 platform with 150-bp paired-end reads at BerryGenomics Company (http://www.berrygenomics.com/. Beijing, China) and Jiangbei New Area Biopharmaceutical Public Service Platform Co., Ltd. (Nanjing, China). The raw data were trimmed with Cutadapt to remove adapters and low-quality bases. Bowtie 2 [50] was used to match reads to the *Arabidopsis* genome TAIR10. After mapping, only unique reads were used for analysis in the next step. 4acC peaks were called by MASC2 [39] with the cutoff of a false discovery rate (FDR) < 0.05. 4acC peaks overlapping between two biological replicates of a sample were retained for further analyses. Metagene plots of genes were generated by calculating the mean 4acC signals in 20 bins of equal length in the generic region from the transcription start site to the poly-A site as well as the − 1 kb and + 1 kb flanking regions. For differential analysis of 4acC, peaks for the WT and mutant plants were merged together and analyzed using DESeq2 (v.3.22.5). 4acC regions with log2(FC) > 1 and FDR < 0.05 were defined as differentially acetylated regions (DARs).

### Gene expression analysis by RNA-seq

For expression profiling, total RNA was isolated from two biological replicates of 3-week-old *Arabidopsis* Col-0 plants using TRIzol according to the manufacturer’s protocol. The amount and quality of RNA were tested with an Agilent Bioanalyzer 2100 system and a Qubit RNA Assay Kit (Life Technologies). Ribosomal RNA was removed by using an mRNA Miniprep Kit (Sigma, MRN10). An NEB Next Ultra II RNA Library Prep Kit for Illumina (NEB, E7770S) was used to construct the library, and sequencing was performed on an Illumina NovaSeq6000 platform with 150-bp paired-end reads at Annoroad Gene Technology Co., Ltd. (Beijing, China). The clean reads were aligned to TAIR10 using TopHat v2.0.9 [51]. Cufflinks v2.1.1 was used to calculate the FPKMs of the coding genes [52]. Differentially expressed genes (DEGs) were called using Cuffdiff (v2.1.1) with an FDR < 0.05.

### Motif analysis

Consensus motifs containing 4acC peaks were identified by MEME-ChIP [46]. Peak centers plus their upstream and downstream 300-bp sequences were used to identify consensus motifs.

### RNA-seq, bisulfite sequencing, DH-seq, ChIP-seq, and DAP-seq data processing

We downloaded previously published RNA-seq data for *met1*; bisulfite sequencing [42], DH-seq [45], ChIP-seq data for H3K4me2 [53], H3K4me3 [53], H3K36me3 [54], H3K9ac [54], H3K14ac [55], H3K9me2 [53], and H3K27me3 [54]; ChIP-chip data for H3K9me3 [56]; and DAP-seq data [47] for ERF3, ERF11, ERF115, BPC1, ANAC71, DREB2A, MYB63, and RAP2-11. The reads from RNA-seq, bisulfite sequencing, ChIP-seq and DAP-seq were mapped to TAIR10 using Bismark [57] and Bowtie [50], respectively. Heatmaps of different epigenetic modifications were generated by using deepTools 2.0 software [58]. DMRs were identified by using the R/Bioconductor package DSS [59]. The DMLtest and callDMR functions in the DSS package (delta = 0.1, p.threshold = 0.05, minCG = 4, minlen = 100) were used with MethylC-seq data (CG or CHG or CHH sites at ≥ 5× coverage) to determine the significant DMRs.

## Supplementary Information

**Additional file 1: Fig. S1-S7**.

**Additional file 2: Table S1.** A summary of 4acC-IP-seq and RNA-seq information.

**Additional file 3: Table S2.** Confident 4acC peaks identified by two independent 4acC-seq experiments in *Arabidopsis* under normal condition.

**Additional file 4: Table S3.** GO analysis of 4acC-containing expressed genes.

**Additional file 5: Table S4.** GO analysis of non-4acC expressed genes.

**Additional file 6: Table S5.** Confident 4acC peaks identified by two independent 4acC-seq experiments in the *met1* mutant.

**Additional file 7: Table S6.** Confident 4acC peaks identified by two independent 4acC-seq experiments in the *rdd* mutant.

**Additional file 8: Table S7.** DARs identified in *met1* mutant compared to Col-0 wild-type.

**Additional file 9: Table S8.** DARs identified in *rdd* mutant compared to Col-0 wild-type.

**Additional file 10: Table S9.** DMRs identified in *met1* and *rdd* mutants compared to Col-0 wild-type.

**Additional file 11: Table S10.** Consensus motifs associated with 4acC peaks.

**Additional file 12:** Review history.

## Data Availability

All sequencing data generated from this study have been deposited into the NCBI Gene Expression Omnibus under the accession number GSE168538 (https://www.ncbi.nlm.nih.gov/geo/query/acc.cgi?acc = GSE168538) [60]. Other datasets supporting the conclusions of this article are listed as follows. RNA-seq and bisulfite sequencing were obtained from published studies (GEO accession: GSE39901) [42]. DH-seq data were acquired from published studies (GEO accession: GSE34318) [45]. ChIP-seq data were obtained from published studies (GEO accession: GSE28398 and GSE137474; DDBJ accession: DRA005154) [53–55]. ChIP-chip data were obtained from published studies (ArrayExpress accession: E-MEXP-951) [56]. DAP-seq data were acquired from published studies (GEO accession: GSE60143) [47].
