# Supplementary material for: N4-acetyldeoxycytosine DNA modification marks euchromatin regions in Arabidopsis thaliana
Source: Genome Biol. 2022 Jan 3;23:5. doi: 10.1186/s13059-021-02578-7 (PMC8722123; doi:10.1186/s13059-021-02578-7)
Supplement: Supplementary file 1 — Additional file 1: Fig. S1-S7. [file 13059_2021_2578_MOESM1_ESM.pdf]

Additional file 1 (Figure S1 to Figure S7)

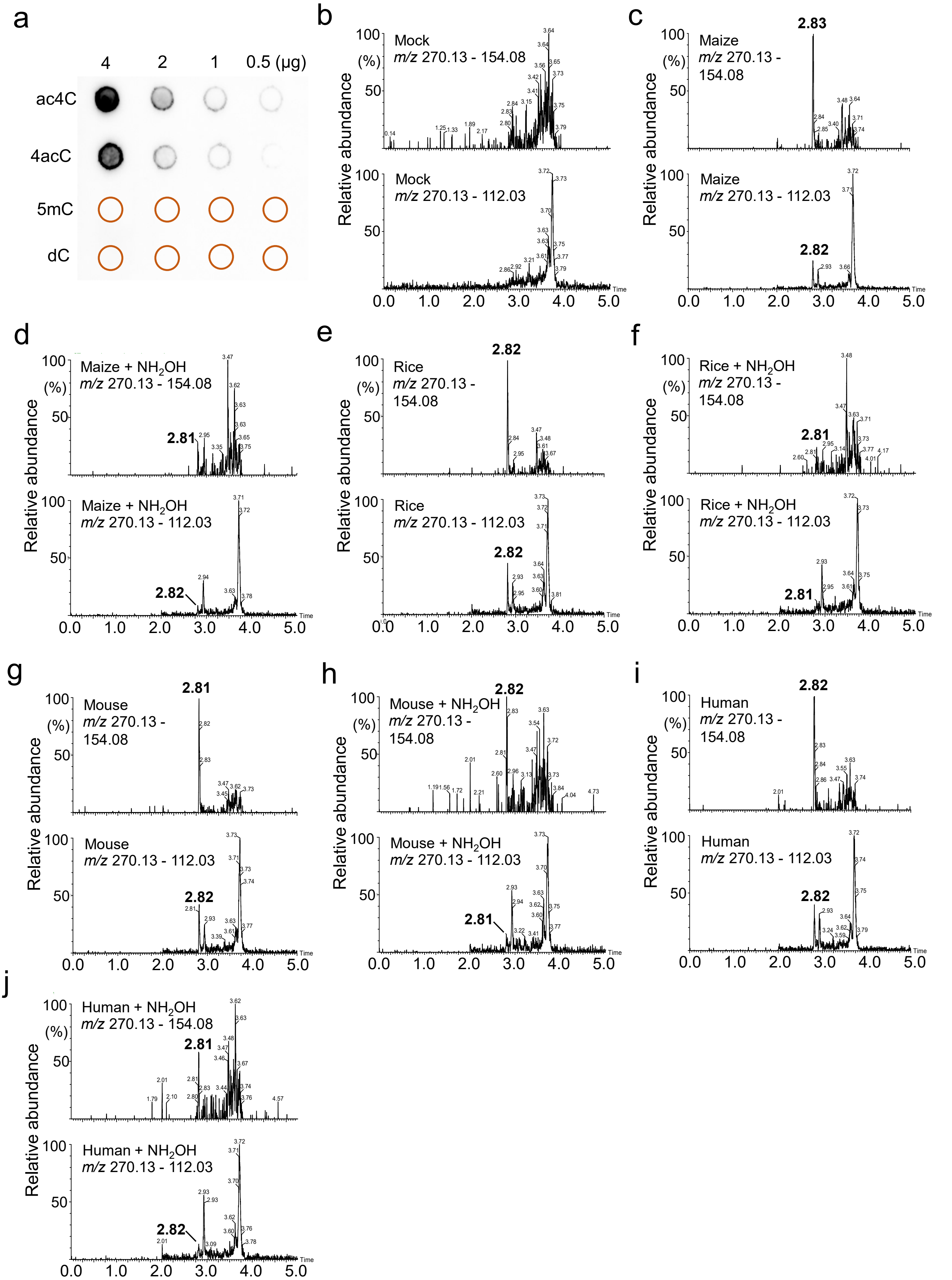

Figure S1: Detection of 4acC-modified nucleosides.

a. Dot blot of 4 nucleosides detected from four amounts by the anti-ac4C antibody. Both 4acC and ac4C could be detected, but 5mC and dC could not. **b.** Detection of 4acC in mock samples by UPLC-ESI-MS/MS. **c-j.** UPLC-ESI-MS/MS chromatograms showing 4acC peaks in gDNA samples without or with NH_2OH treatment from maize (seedlings) (**c and d**), rice (seedlings) (**e and f**), mouse (liver) (**g and h**) and human (293T cells) (**i and j**). The selective multiple reaction monitoring transitions for 4acC were set as m/z 270.13-154.08 and m/z 270.13-112.03. The retention time of standard 4acC was observed in these three gDNA samples.

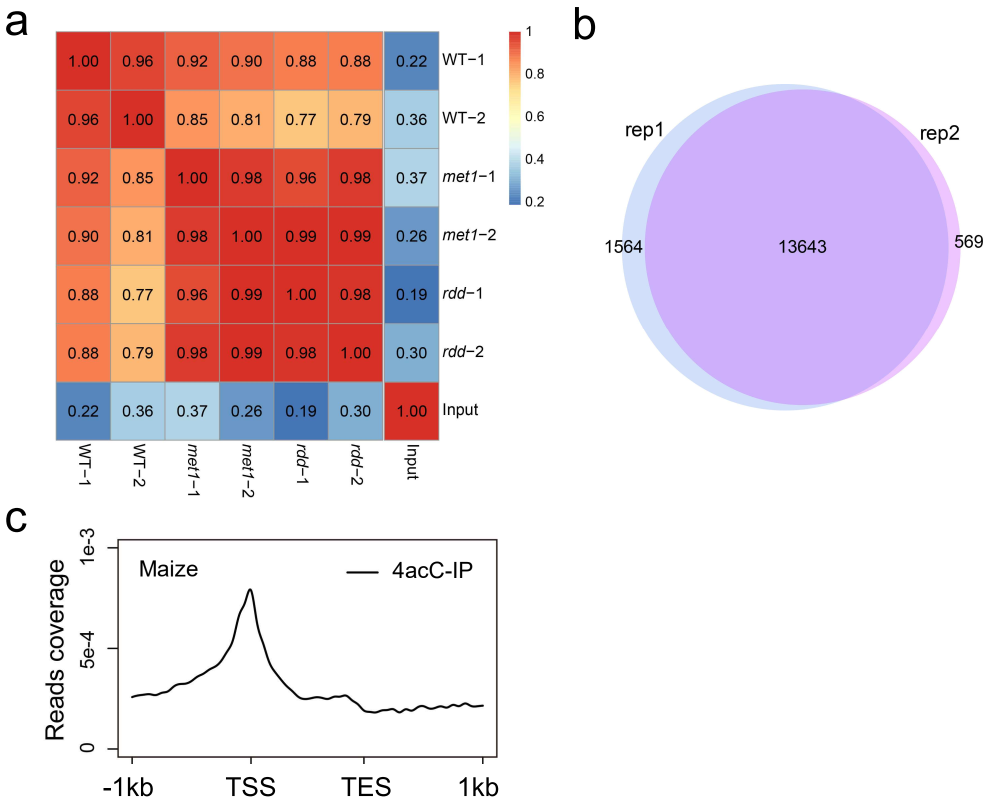

Figure S2: Pearson correlation analysis and peak calling of 4acC-IP samples. **a.** Pearson correlation analysis of reads from 4acC-IP-seq or input-seq in this study. **b.** Overlaps of 4acC peaks in two biological samples, indicating high reproducibility. R, Pearson correlation coefficient; Rep, Replicate. **c**. Distribution of 4acC peaks over protein-coding gene regions in maize. The 1 kb upstream and downstream flanking coding regions were aligned for all genes.

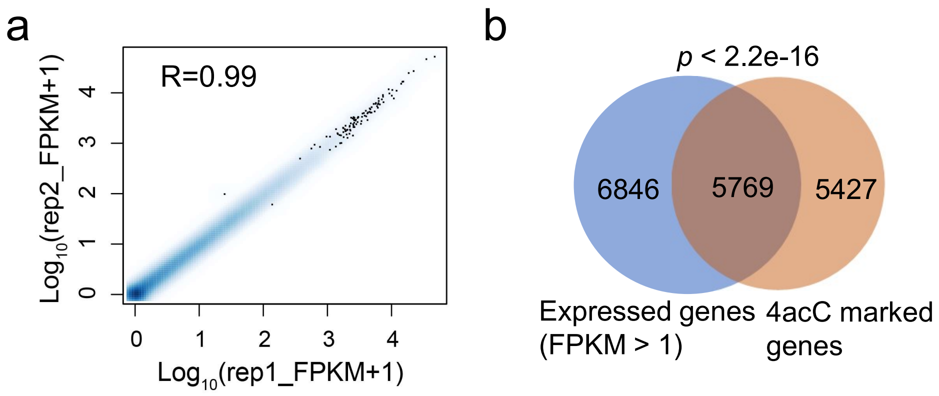

Figure S3: Association of 4acC with gene expression.

a. Pearson correlation analysis of RNA-seq reads in two biological replicates. RNA-seq experiments were performed with two independent biological replicates from 3-week-old WT plants under normal (22 °C) temperature. R, Pearson correlation coefficient; Rep, Replicate. **b.** Venn diagram showing the overlaps between expressed genes (FPKM > 1) and 4acC-marked genes. The *p* values were calculated by Fisher's exact test.

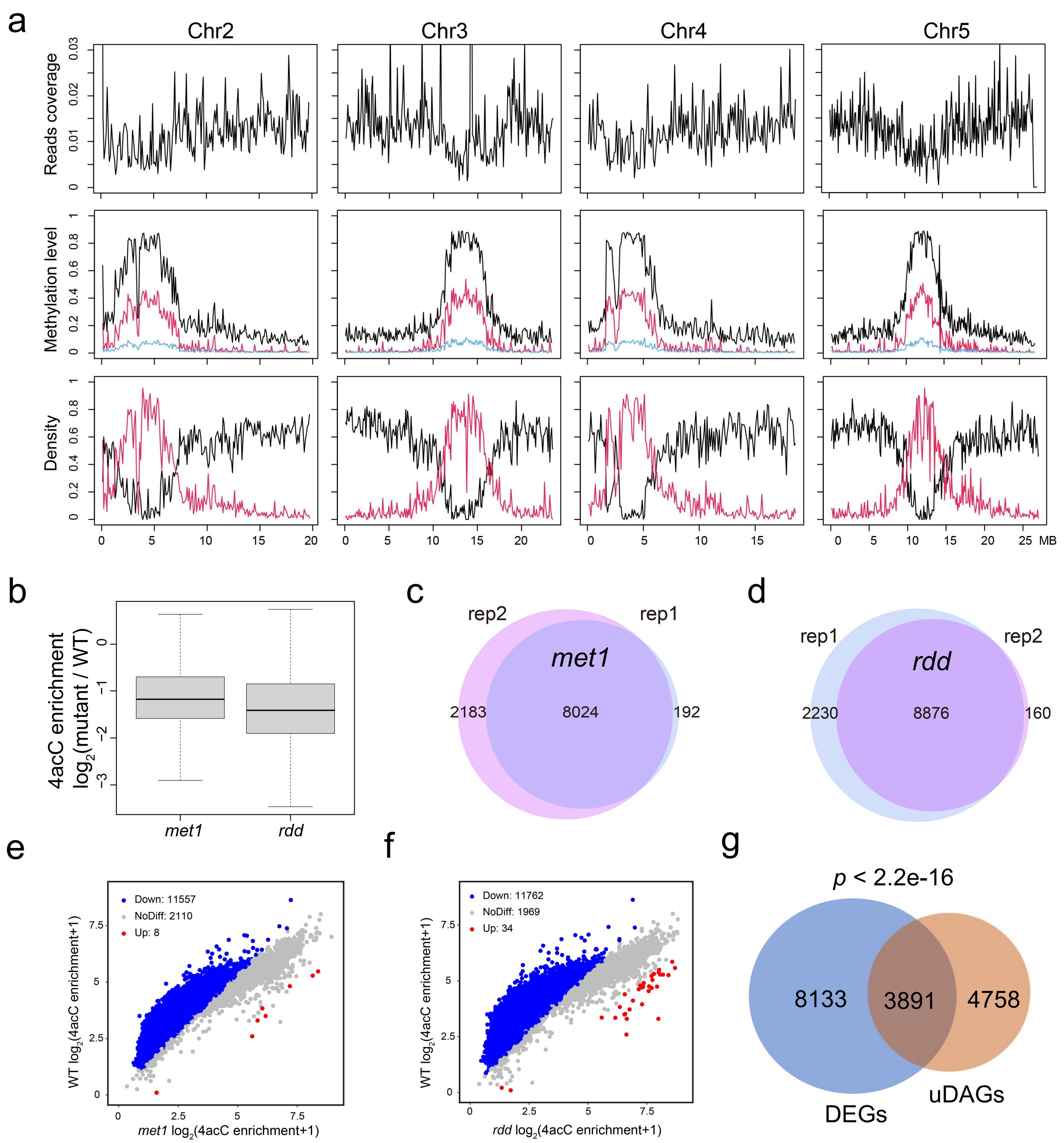

Figure S4: Identification and analysis of 4acC peaks in *met1* and *rdd* mutants.

a. 4acC distribution in *Arabidopsis* chromosomes 2-5. The top panel shows the average 4acC levels per 100 kb bin. The middle panel shows the average 5mC levels at CG, CHG and CHH sites, and the bottom panel shows the densities of genes and TEs. **b.** Box plots of 4acC abundance in *met1* and *rdd* mutants versus WT plants. **c, d.** Venn diagram showing the overlaps of 4acC peaks between two biological replicates in *met1* (**c**) and *rdd* (**d**) mutants. **e**, **f.** Scatter plots showing the peaks with differential 4acC levels ($|\log_2 FC| \geq 1$, FDR < 0.05) in *met1* (**e**) and *rdd* (**f**) mutants versus WT plants. **g.** Overlaps of DEGs with uDAGs in the *met1* mutant compared to the WT. The *p* values were calculated by Fisher's exact test.

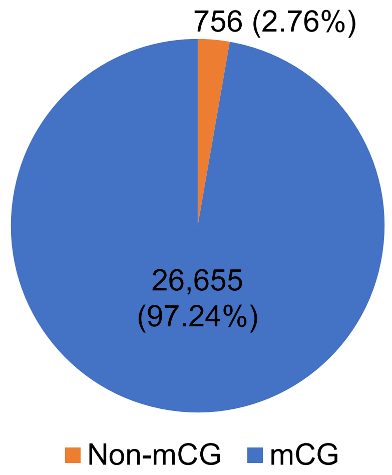

Figure S5: Percentages of genes with or without 5mC (mCG) in *Arabidopsis*. Numbers and percentages of non-mCG- and mCG-methylated genes within coding regions in *Arabidopsis*.

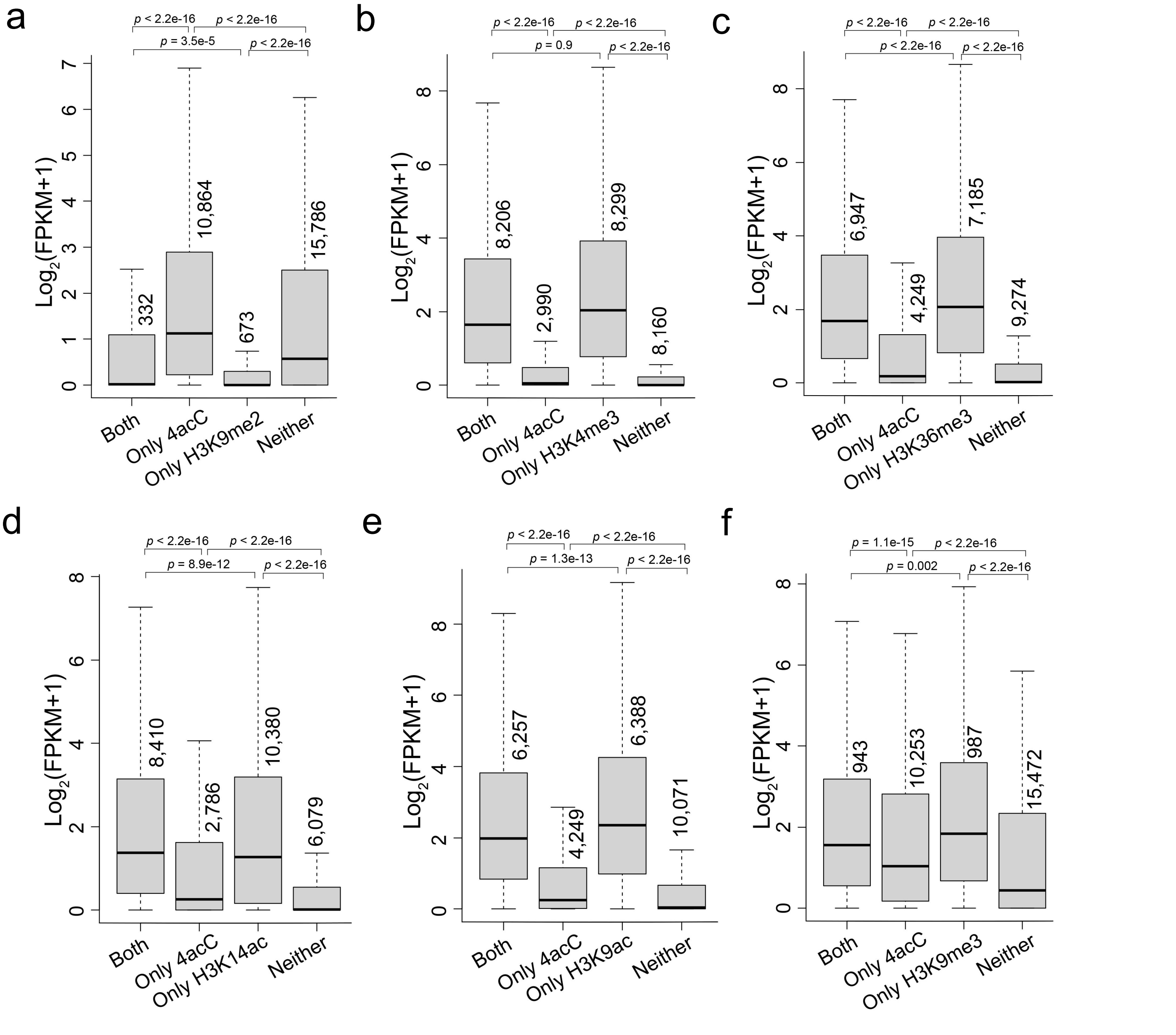

Figure S6: Interactions between 4acC and histone modification marks in gene expression regulation.

a-f. Box plot of the expression levels of a group of genes marked or not marked by H3K9me2 (**a**), H3K4me3 (**b**), H3K36me3 (**c**), H3K14ac (**d**), H3K9ac (**e**) or H3K9me3 (**f**) with or without 4acC. The p values were calculated for significant differences between two groups by the Mann–Whitney U test.

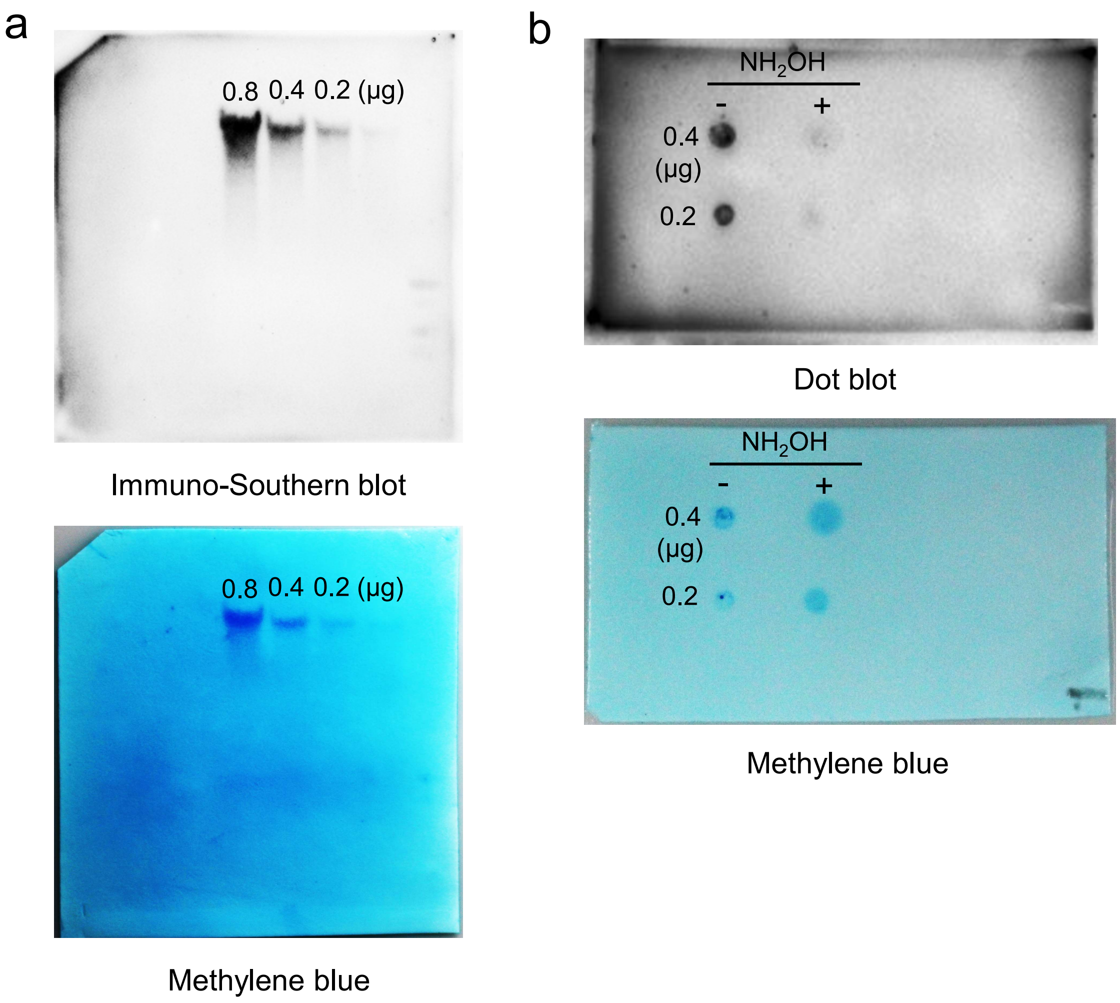

Figure S7: Full images for Fig. 1a and b.

Anti-4acC specific antibody was used to detect 4acC signals in gDNA extracted from 3-week-old Col-0 WT plants. The amount of gDNA loaded is indicated. **a.** Full Immuno-Southern blot images for Fig. 1a. **b.** Full dot-blot images for Fig. 1b.
