# Supplementary material for: N4-acetyldeoxycytosine DNA modification marks euchromatin regions in Arabidopsis thaliana
Source: Genome Biol. 2022 Jan 3;23:5. doi: 10.1186/s13059-021-02578-7 (PMC8722123; doi:10.1186/s13059-021-02578-7)
Supplement: Supplementary file 12 — Additional file 12: Review history. [file 13059_2021_2578_MOESM12_ESM.docx]

Review History

**First round of review**

**Reviewer 1**

**Are you able to assess all statistics in the manuscript, including the appropriateness of statistical tests used?**

**Yes, and I have assessed the statistics in my report.**

**Comments to author:**

This is the review of the manuscript "N4-acetyldeoxycytosine DNA modification marks euchromatin regions in Arabidopsis thaliana" submitted by Wang and colleagues to Genome Biology.

They applied different methods to validate that 4acC is present in the genomic DNA of Arabidopsis thaliana. 4acC-IP-seq was preformed to generate genome-wide profiling of 4acC. The 4acC-IP-seq analysis revealed that 4acC peaks are distributed in euchromatin. By integrating multiple epigenomic datasets and gene expression datasets, they found that 4acC is potential associated with gene expression regulation. The distribution of 4acC is distinct with the typical 5mC. Moreover, they also found that 4acC is associated with active histone modifications.

This is a well conducted and time-consuming study, which has a potential to attract attentions of researchers that study epigenetics and gene expression regulation. The annotation of 4acC will be an important resource for researchers in plants and epigenetics. The analyses were conducted in a logic way, but sometimes there are trivial grammar mistakes, indicating that the writing need to be improved. It is suitable to publish in a high impacted journal like GB after the following points have been addressed.

Major point:

After reading this article, I have a feeling that a lot of analysis are predictable to a certain extent. For example, 4acC shows positive correlation with gene expression, so the relationship with 5mC and active histone modification can be expected. It needs to be done by altering the level of 4acC, to check whether 4acC is the cause of gene differential expression. Additionally, for a new epigenetic modification, it is more important to find the reader, writer, or eraser. Only in that way could really show the importance of this modification.

Moreover, the authors have also done a lot of experiments and analysis in rice and maize, but there is not much related discussion in this paper.

Lastly, regarding the structure of the discussion section, the authors should move results (L320-332) to results section.

Minor point:

* There are a lot of grammar mistakes in the manuscript, which needs to be checked carefully.

* In Fig. 2d, kb should be used.

* In L695, should it be Fisher's Exact Test?

* In Fig. S4a-b, no legend, and no test.

* Like 5mC and m5C, m6A and 6mA, the authors should unify the writing standard.

* For comparisons among three and more groups, Kruskal-Wallis test or subsequent Mann-Whitney U test is better.

* In methods part: L424, L433, L444 are about the NGS analysis. The author's analysis method of these data is too simple and should be written clearly and have more details of the analysis.

* The scripts and code used in the analyses should be provided in a public repository, e.g., Github.

* L170-L172: Is the parameter of gene expression too low? More tests should be provided.

* L174: It should be the AgriGO V2 but not V1 based on the reference.

* L198: The authors should classify which allele of met1 is used.

* L285: "Specific chromatin states determine the patterns of gene activity and gene silencing" is an over-statement.

**Reviewer 2**

**Are you able to assess all statistics in the manuscript, including the appropriateness of statistical tests used?**

No, I do not feel adequately qualified to assess the statistics.

**Comments to author:**

This is quite a striking discovery. They authors detected a large amount of 4acC in plant genomic DNA. Based on dot blot and mass spectrometry measurement the level could be 1-2% of all C in plant genomic DNA. They also performed 4acC IP followed by sequencing. Results make sense that this modification seems to be preferrentially installed onto active genes or at ssDNA stage (the 4 position of C is hindered in duplex DNA). The mark appears to correlate with gene activation. The claim is provocative. I have a number of questions that need to be addressed:

1. Fig 1c. The level of 4acC is too high. 5mC marks constitutive and facultative heterochromatin which compose most of genome. 4acC marks euchromatin which should be a small portion of the genome. It makes no sense 4acC is so abundant if it only marks active genes. Either the measurement is wrong or the samples severely contaminated with other species. The authors can sequence the exact same sample to look for contamination. In their IP experiment did they observe 4acC peaks not mapped to plant genome? These are likely contaminates.

2. Fig. 2a showed modest enrichment of 4acC, again arguing against high abundance reported by the authors.

3. Can the authors detect any motif that enrich 4acC. I think it is critical to detect a motif and use restriction enzyme digestion to at least obtain modification fraction at a few sites. The authors can use base treatment as a control. A few sites with high 4acC will be key evidence.

4. On Fig. 6b can the authors also show correlation with K9me3?

5. For DNA 4acC, its mass spec standard should show strong intensity in 2 channels, as in both 270.1 --> 112 and 270.1 --> 154. In the current version, the authors only showed 4acC signals in channel 270.1 --> 112. The author should provide mass spec images for all biological samples in these 2 channels, with a major peak in each channel expected.

6. The author showed gDNA 4acC mass spec peak of rice, maize, HEK293T and mouse liver in Figure S1d. However, the image only covered 3.5 min ~ 4.25 min, with marking of the 4acC peak at 3.76 min. The full image from 0 min to 5 min should be shown here.

7. The author used hydroxylamine to remove 4acC when performing the sequencing. As the confirmation for Figure S1d, for gDNA 4acC in rice, maize, HEK293T and mouse liver, mass spec image showing how 4acC peak is removed after hydroxylamine treatment should be shown.

**Authors Response**

**Point-by-point responses to the reviewers’ comments:**

Reviewer #1:

This is the review of the manuscript "N4-acetyldeoxycytosine DNA modification marks euchromatin regions in Arabidopsis thaliana" submitted by Wang and colleagues to Genome Biology.

They applied different methods to validate that 4acC is present in the genomic DNA of Arabidopsis thaliana. 4acC-IP-seq was preformed to generate genome-wide profiling of 4acC. The 4acC-IP-seq analysis revealed that 4acC peaks are distributed in euchromatin. By integrating multiple epigenomic datasets and gene expression datasets, they found that 4acC is potential associated with gene expression regulation. The distribution of 4acC is distinct with the typical 5mC. Moreover, they also found that 4acC is associated with active histone modifications.

This is a well conducted and time-consuming study, which has a potential to attract attentions of researchers that study epigenetics and gene expression regulation. The annotation of 4acC will be an important resource for researchers in plants and epigenetics. The analyses were conducted in a logic way, but sometimes there are trivial grammar mistakes, indicating that the writing need to be improved. It is suitable to publish in a high impacted journal like GB after the following points have been addressed.

*Response: We thank the reviewer for the comments. We also used Springer Nature Author Services to improve the writing.*

Major point:

After reading this article, I have a feeling that a lot of analysis are predictable to a certain extent. For example, 4acC shows positive correlation with gene expression, so the relationship with 5mC and active histone modification can be expected. It needs to be done by altering the level of 4acC, to check whether 4acC is the cause of gene differential expression. Additionally, for a new epigenetic modification, it is more important to find the reader, writer, or eraser. Only in that way could really show the importance of this modification. Moreover, the authors have also done a lot of experiments and analysis in rice and maize, but there is not much related discussion in this paper. Lastly, regarding the structure of the discussion section, the authors should move results (L320-332) to results section.

*Response: Thanks for the suggestions. Our prior analysis for the relationship between 4acC and 5mC DNA modification showed that the global 4acC abundance was decreased in both met1 and rdd mutants compared to WT. To investigate the alteration of 4acC level affecting on gene expression, we analyzed the overlaps between differentially expressed genes (DEGs) and unique differentially acetylated genes (uDAGs) in met1 mutant compared to WT. The uDAGs were identified as DAGs that were not overlapped with differentially methylated genes (DMGs). RNA-seq data of met1 mutant from previous study (Stroud et al., 2013) and WT were used to analyze change of gene expression. We found that 45% of uDAGs showed expression level changes and 36% of DEGs overlapped with uDAGs in met1 mutant, suggesting 4acC plays a role in gene expression regulation. The revised manuscript added these data in L223-L230.*

*In addition, we agree your opinion about the writer/eraser, which will be aim of our study in future. We are performing experimental verifications after we predicted some candidate genes.*

*We conducted UPLC-ESI-MS/MS detection of 4acC in rice and maize, and performed once 4acC-IP-seq experiment for maize in our studies. As the reviewer suggested, we added discussion contents in the revised manuscript (L309-L311).*

*We moved this paragraph to the results section in the revised version (L292-L304).*

Minor point:

1. There are a lot of grammar mistakes in the manuscript, which needs to be checked carefully.

*Response: We are very sorry for the grammar mistakes in the manuscript. This revised manuscript has been edited and proofread by a professional language editing service.*

2. In Fig. 2d, kb should be used.

*Response: Correction has been made in the revised version.*

3. In L695, should it be Fisher's Exact Test?

*Response: Yes, we actually used Fisher's Exact Test in that analysis. We are very sorry for the description error. It has been corrected in the revised version. Thanks.*

4. In Fig. S4a-b, no legend, and no test.

*Response: We added figure legends in the revised version. In Fig. S4b, we tried to show the general and genome-wide decrease of 4acC abundance in met1 and rdd mutants compared to WT. We did not compare the proportion of decrease between the mutants. This the reason we did not make the test.*

5. Like 5mC and m5C, m6A and 6mA, the authors should unify the writing standard.

*Response: Thanks for the suggestion. 5mC/m5C, or 6mA/m6A are direct analogs of chemically modified bases that occurred on DNA and RNA, respectively. To avoid ambiguity and misunderstanding, we defined these abbreviations in more detail and unified the writing standard in the revised manuscript (Line 25-27).*

6. For comparisons among three and more groups, Kruskal-Wallis test or subsequent Mann-Whitney U test is better.

*Response: The full name of Wilcoxon test used in our analysis is actually Mann-Whitney U test. We unified the use of Mann-Whitney U test in the revised manuscript.*

7. In methods part: L424, L433, L444 are about the NGS analysis. The author's analysis method of these data is too simple and should be written clearly and have more details of the analysis.

*Response: Thanks for the suggestion. We have added the details (Line 442-448, 457-459).*

8. The scripts and code used in the analyses should be provided in a public repository, e.g., Github.

*Response: We put the scripts and codes in https://github.com/meffey/NGS_Chip_code.*

9. L170-L172: Is the parameter of gene expression too low? More tests should be provided.

*Response: We further analyzed the overlaps between 4acC-containing genes and expressed genes that were with a FPKM > 1. This analysis revealed that 46% of expressed genes contain 4acC modification, and 51% of the 4acC marked genes show expressions. The results were added in the revised manuscript (Fig. S3b, Line 172-177).*

10. L174: It should be the AgriGO V2 but not V1 based on the reference.

*Response: Thanks. We used AgriGO V2 for GO enrichment analysis in this study.*

11. L198: The authors should classify which allele of met1 is used.

*Response: We used met1-3 mutant in this study. The details were marked in materials and methods section (L362).*

12. L285: "Specific chromatin states determine the patterns of gene activity and gene silencing" is an over-statement.

*Response: The statement “Specific chromatin states determine the patterns of gene activity and gene silencing” was changed to “Specific chromatin states are associated with the patterns of gene activity and gene silencing” in the revised version (Line 315).*

Reviewer #2:

This is quite a striking discovery. They authors detected a large amount of 4acC in plant genomic DNA. Based on dot blot and mass spectrometry measurement the level could be 1-2% of all C in plant genomic DNA. They also performed 4acC IP followed by sequencing. Results make sense that this modification seems to be preferentially installed onto active genes or at ssDNA stage (the 4 position of C is hindered in duplex DNA). The mark appears to correlate with gene activation. The claim is provocative. I have a number of questions that need to be addressed:

*Response: We thank the reviewer for the comments, especially for the UPLC-ESI-MS/MS, which gave us the chance to learn the details and improve the results.*

1. Fig 1c. The level of 4acC is too high. 5mC marks constitutive and facultative heterochromatin which compose most of genome. 4acC marks euchromatin which should be a small portion of the genome. It makes no sense 4acC is so abundant if it only marks active genes. Either the measurement is wrong or the samples severely contaminated with other species. The authors can sequence the exact same sample to look for contamination. In their IP experiment did they observe 4acC peaks not mapped to plant genome? These are likely contaminates.

2. Fig. 2a showed modest enrichment of 4acC, again arguing against high abundance reported by the authors.

*Response to 1 and 2: Thanks for the critical comments. The reads of 4acC-IP-seq experiments that used the same gDNA samples with UPLC assay showed high mapping ratio (>92%), suggesting no contaminates. To further verify the reliability of 4acC level measured in our prior analysis, we freshly extracted gDNA samples from Arabidopsis, and applied a more accurate UPLC-ESI-MS/MS assay to absolutely quantify 4acC level. The 4acC level is about 0.1% of dC. The lower level of 4acC measured by UPLC-ESI-MS/MS than previous UPLC assay may be caused by the different detection methods. We replaced the original data of 4acC level with the latest and more accurate results in the revised version.*

*Furthermore, we compared the genomic coverage of 5mC and 4acC. By analysis of 5mC-IP-seq data from previous reports (Zhang et al., 2006), we identified 26,852 peaks that cover 22.6 M genomic regions. In the analysis of 4acC-IP-seq, we identified 13,643 peaks that represent 10.5 M genomic regions. The modification regions of 5mC are about twice that of 4acC according to the IP-seq data. By single-base resolution identification and mapping of 5mC, we knew that 5mC distributed in most of the genome. Therefore, the development of high-resolution methods in the future for detecting 4acC may expand the distribution ranges of 4acC.*

3. Can the authors detect any motif that enrich 4acC. I think it is critical to detect a motif and use restriction enzyme digestion to at least obtain modification fraction at a few sites. The authors can use base treatment as a control. A few sites with high 4acC will be key evidence.

*Response: Yes, we searched for consensus motifs using MEME-ChIP. The top two significantly enriched motifs were CDYCDYCDYCDYCDY (D represents A, G and T; Y represents C and T; E-value = 5.3×10-152) and YCTCTCTYTCTYYYT (E-value = 3.9×10-74), both of which were not intersected with CG, CHG or CHH. It is true as the reviewer commented that utilization of restriction enzyme to verify some of 4acC sites will be pivotal evidence to prove the existence and distribution of 4acC, like the use of methylation-sensitive restriction endonuclease in examining 5mC and 6mA sites. However, we have not found a restriction enzyme that can distinguish cytosine with or without acetylation yet. We will continue to find ways to test 4acC sites in the following work (Line 292-304).*

4. On Fig. 6b can the authors also show correlation with K9me3?

*Response: We added the correlation analysis of 4acC with H3K9me3 in Fig. 6b in the revised version.*

5. For DNA 4acC, its mass spec standard should show strong intensity in 2 channels, as in both 270.1 --> 112 and 270.1 --> 154. In the current version, the authors only showed 4acC signals in channel 270.1 --> 112. The author should provide mass spec images for all biological samples in these 2 channels, with a major peak in each channel expected.

*Response: Thanks a lot! It is true as the reviewer said that the mass spec of 4acC standard indeed shows strong intensity in 2 channels, 270.1 → 154.08 and 270.1 → 112.03. We provided the data in these 2 channels for all biological samples in the revised manuscript (Line 120-129). Please check the 2nd paragraph in this letter for more details.*

6. The author showed gDNA 4acC mass spec peak of rice, maize, HEK293T and mouse liver in Figure S1d. However, the image only covered 3.5 min ~ 4.25 min, with marking of the 4acC peak at 3.76 min. The full image from 0 min to 5 min should be shown here.

*Response: We provided the full image from 0 min to 5 min in the revised manuscript.*

7. The author used hydroxylamine to remove 4acC when performing the sequencing. As the confirmation for Figure S1d, for gDNA 4acC in rice, maize, HEK293T and mouse liver, mass spec image showing how 4acC peak is removed after hydroxylamine treatment should be shown.

*Response: We examined 4acC in hydroxylamine treated gDNA samples from Arabidopsis, rice, maize, human and mouse by UPLC-ESI-MS/MS, and found that the relative abundance of 4acC was obviously decreased. We provided these data in the Fig. S1 of revised manuscript.*

**Second round of review**

**Reviewer 1**

The authors addressed all of my previously concerns well. Further minor issues are detailed below, which could be easy to address.

1. L31: Authors should describe more accurately: “we demonstrated the existence of N4-acetyldeoxycytosine (4acC) in genomic DNA (gDNA) of Arabidopsis, rice, maize, mouse and human with multiple detection methods”. They only applied “multiple” methods to detect and quantify the 4acC in Arabidopsis, maize and rice.

2. L259: The format of “Thirty-two percent” and 57% should be unified. 3. The format of unit needs to be unified. For example, L751: “1 kb” and L752: “1-kb”.

**Reviewer 2**

We checked our previous mass spec results for mammalian cells and tissues. We did not see this peak from mammalian systems. Otherwise I am sure people would have reported. It is too obvious for people to miss. For plant it could be a different story as I do not know how extensively plant DNA has been subjected to careful mass spec analysis. I am still not confident about the identity of the peak. Here are two experiments I think the authors need to perform at least one to convincingly show the present of this new modification.

1. Isolate plant DNA, digest to single nucleotide, label with P32, run TLC and 2-D TLC to compare with a synthetic standard. I think this might be the easiest.

2. Isotope label the acetyl group from the biological sample and show with mass spec correct shift. Challenge is to grow plants with isotope labeled acetyl.

I would strongly suggest remove mammalian data. Do not think it is there unless the authors can also do 1 or 2 in mammals as well.

**Authors Response**

**Point-by-point responses to the reviewers’ comments:**

Reviewer #1:

The authors addressed all of my previously concerns well. Further minor issues are detailed below, which could be easy to address.

1. L31: Authors should describe more accurately: “we demonstrated the existence of N4-acetyldeoxycytosine (4acC) in genomic DNA (gDNA) of Arabidopsis, rice, maize, mouse and human with multiple detection methods”. They only applied “multiple” methods to detect and quantify the 4acC in Arabidopsis, maize and rice.

*Response: We thank the reviewer for the comments. We changed the statements in the revised version.*

2. L259: The format of “Thirty-two percent” and 57% should be unified.

*Response: We unified the format in the revised version.*

3. The format of unit needs to be unified. For example, L751: “1 kb” and L752: “1-kb”.

*Response: We unified the format in the revised manuscript.*

Reviewer #2:

We checked our previous mass spec results for mammalian cells and tissues. We did not see this peak from mammalian systems. Otherwise, I am sure people would have reported. It is too obvious for people to miss. For plant it could be a different story as I do not know how extensively plant DNA has been subjected to careful mass spec analysis. I am still not confident about the identity of the peak. Here are two experiments I think the authors need to perform at least one to convincingly show the present of this new modification.

*Response: We thank the reviewer for the rigor and comments. You mentioned that your previous mass spec did not identify the 4acC peak in mammalian systems. We think it may be caused by the difference of experimental conditions or parameters used for mass spec. We sent our samples to a professional and independent platform for UPLC-MS/MS which had supported lots of studies in Nanjing Agricultural University. In addition, the consistent results of immuno-blot and IP-seq supported the reliability of our UPLC-MS/MS system.*

1. Isolate plant DNA, digest to single nucleotide, label with P32, run TLC and 2-D TLC to compare with a synthetic standard. I think this might be the easiest.

*Response: Thanks for your suggestion. 2D-TLC is an intuitive method to detect and quantify nucleotide modification. In this experiment, the 5' end of the digested gDNA fragments and synthetic oligonucleotides containing 4acC modification should be firstly labelled with 32P, and then they will be digested to monophosphonucleotides before 2D-TLC analysis. However, 32P cannot be labelled onto a single 4acC nucleoside standard by polynucleotide kinase. We also consulted with IDT and Genscript Inc. about synthesis of oligonucleotides containing 4acC modification, but they currently unable to provide this service. We are very sorry for not being unable to perform this experiment, owing to technical limitation.*

2. Isotope label the acetyl group from the biological sample and show with mass spec correct shift. Challenge is to grow plants with isotope labeled acetyl.

*Response: Thanks for the suggestion. In this experiment, plants need to be grown in the medium that containing isotope labelled acetyl group, and then gDNA isolated form these plants will be used for further UPLC-MS/MS analysis. Considering the potential danger of radiation, we do not yet have the experimental conditions/facility to protect against radiation at so many procedures among this experiment. We are very sorry for cannot perform this experiment, because of the experimental condition limitations.*
